# Plasma metabolomics reveals lower carnitine concentrations in overweight Labrador Retriever dogs

**DOI:** 10.1186/s13028-019-0446-4

**Published:** 2019-02-26

**Authors:** Josefin Söder, Katja Höglund, Johan Dicksved, Ragnvi Hagman, Hanna Eriksson Röhnisch, Ali Ata Moazzami, Sara Wernersson

**Affiliations:** 1 Department of Anatomy, Physiology and Biochemistry, Faculty of Veterinary Medicine and Animal Science, Swedish University of Agricultural Sciences, Box 7011, 75007 Uppsala, Sweden; 2 Department of Animal Nutrition and Management, Faculty of Veterinary Medicine and Animal Science, Swedish University of Agricultural Sciences, Box 7024, 75007 Uppsala, Sweden; 3 Department of Clinical Sciences, Faculty of Veterinary Medicine and Animal Science, Swedish University of Agricultural Sciences, Box 7054, 75007 Uppsala, Sweden; 4 Department of Molecular Sciences, Faculty of Natural Resources and Agricultural Sciences, Swedish University of Agricultural Sciences, Box 7015, 75007 Uppsala, Sweden

**Keywords:** Canine, Carnitine insufficiency, Meal-challenge test, Metabolic variations, NMR, Nuclear magnetic resonance, Obesity, Postprandial metabolism

## Abstract

**Background:**

The prevalence of overweight is increasing in dogs, but the metabolic events related to this condition are still poorly understood. The purpose of the study was to investigate the postprandial response of plasma metabolites using a meal-challenge test and to identify metabolic variations related to spontaneous overweightness in privately owned dogs.

**Results:**

Twenty-eight healthy male intact Labrador Retriever dogs were included, 12 of which were classified as lean (body condition score (BCS) 4–5 on a 9-point scale) and 16 as overweight (BCS 6–8). After an overnight fast (14–17 h), blood samples were collected and dogs were thereafter fed a high-fat meal. Postprandial blood samples were collected hourly four times. Plasma metabolites were identified by nuclear magnetic resonance. Postprandial metabolomes differed from the fasting metabolome in multivariate discriminant analysis (PLS-DA: Q^2^Y = 0.31–0.63, cross-validated ANOVA: P ≤ 0.00014) Eleven metabolites, all amino acids, contributed to the separations. Carnitine was identified as a metabolite related to overweight (stepwise logistic regression analysis P ≤ 0.03) and overweight dogs had overall lower carnitine response (mixed model repeated measures analysis P = 0.005) than lean dogs. Notably, mean fasting carnitine concentration in overweight dogs (9.4 ± 4.2 µM) was close to a proposed reference limit for carnitine insufficiency.

**Conclusions:**

A postprandial amino acid response was detected but no time-dependent variations with regards to body condition groups were found. Lower carnitine concentrations were found in overweight compared to lean dogs. The latter finding could indicate a carnitine insufficiency related to spontaneous adiposity and altered lipid metabolism in overweight dogs in this cohort of otherwise healthy Labrador Retrievers.

**Electronic supplementary material:**

The online version of this article (10.1186/s13028-019-0446-4) contains supplementary material, which is available to authorized users.

## Background

Obesity is a complex disorder and has become one of the major health concerns in dogs as well as in people [1–4]. Weight management in dogs is challenging for both owners and veterinarians. Current therapies for overweight dogs focus mainly on energy restriction, which is not always successful [5, 6]. Consequently, a large number of dogs suffer from chronic overweightness and have an increased risk of poor quality of life, early onset of chronic diseases, and a shortened lifespan [7–9].

In dogs, as in people, being overweight might result in metabolic disorders, e.g. insulin resistance and hyperlipidaemia [10–13]. Despite the increasing prevalence of obesity among dogs, the molecular mechanisms associated with this condition are still not fully understood. Nuclear magnetic resonance (NMR) is a powerful and widely used tool for identifying molecular changes under various conditions [14]. The NMR technique enables identification and quantification of a variety of metabolites (mainly small amines and organic acids) and might provide new insights into metabolism in health and disease. Recent metabolomics studies have revealed that overweight humans often have elevated concentrations of plasma acylcarnitines [15–17] and branched-chain amino acids [18, 19] and that overfed dogs might show a biphasic pattern of various plasma metabolites, i.e. an increase during acute weight gain, and later a normalization in chronic overweight [20]. The NMR technique has the potential to identify metabolic variations between lean and overweight dogs, but has not been widely used to study plasma metabolites in spontaneously overweight dogs.

In the few studies investigating fasting plasma metabolomes in dogs, the fasting metabolome has been shown to vary depending on individual variations and diet [20–22] and overweight dogs display variations in metabolites related to lipid metabolism [23, 24]. In humans, the postprandial metabolome is less variable than fasting and can generate more information about important metabolic changes that would otherwise be difficult to detect [25–29]. In overweight dogs, we have previously found more pronounced metabolic variations in postprandial compared to fasting urine [30]. Therefore, plasma metabolomics in combination with a meal challenge has the potential to enhance the understanding of canine metabolism and to detect subtle metabolic variations related to overweightness in dogs. To the best of our knowledge, the plasma metabolome of lean and spontaneously overweight, but otherwise healthy dogs, during a meal-challenge test has not previously been published. Therefore, the aim of the study was to investigate postprandial plasma metabolites in lean and overweight healthy Labrador Retrievers using a meal-challenge test and to identify metabolic variations related to spontaneous overweightness.

## Methods

### Animals

The study population consisted of 28 privately-owned intact male show-type Labrador Retriever dogs. To qualify for inclusion in the study, each dog had to be considered healthy by its owner, pass the health examination outlined below and have had a stable body weight for at least 3 months. Exclusion criteria consisted of historic or current systemic or organ-related diseases or treatment with; antibiotics, non-steroid anti-inflammatory drugs, steroids, deworming drugs or proton pump inhibitors within 3 months prior to the examination day. Dogs were also excluded if vital parameters, haematology or serum biochemistry were outside reference ranges for healthy dogs on the day of examination. Dogs were recruited by a personal letter to 715 owners (mostly located within 100 kilometres of Uppsala, Sweden) of potentially eligible male Labrador Retrievers registered by The Swedish Kennel Club. Recruitment and data collection were performed within a 1-year period and dogs were sampled once at the same time of the day according to a pre-designed protocol. Sixty owners replied and their dogs were examined for eligibility by an on-line survey of the dogs’ health status and their feeding and exercise routines. Thirty-two dogs were not invited for further data collection, based on information stated in the online surveys and one dog owner declined further participation (dog number 4). The remaining 28 dogs were invited to participate in the data collection of the meal-challenge test. No dog was excluded during sample collection and there were no missing data for the 28 included dogs. All included dogs were of show line in five generations according to an expert at The Swedish Kennel Club.

### General study design

Dietary history was acquired by daily food diaries provided by the dog owners during 2 weeks preceding the study. According to the food diaries, all dogs had their main energy supply from dry or wet complete commercial diets and the most common protein source in the complete diets was chicken. A limited number of dogs were fed a low-fat calorie-restricted diet, obtained their main metabolizable energy from fat, or were given complete diets containing l-carnitine additives (Table 1). No adjustments were made to the dogs’ regular home diet of complete commercial dog foods and treats prior to participation in the study. The dogs were fasted in their home environment from 6 pm on the day before the clinical samplings. In the morning of the examination day, water was withheld and a voided urine sample was taken from each dog by the owner. On arrival at the clinic (between 8 and 9:30 am, after 14–17 h of fasting), the dogs were examined by the same veterinarian (JS), fasting blood samples were taken, and the test meal was provided. After meal feeding, blood samples were collected hourly four times. The study was performed at the Swedish University of Agricultural Sciences, Uppsala, Sweden and was approved by the Ethical Committee for Animal Experiments, Uppsala, Sweden (C180/12). The owner’s written consent was obtained for all dogs. This prospective study followed guidelines for reporting observational studies in epidemiology [31].

**Table 1:** Background diet in home environment of the 28 Labrador Retriever dogs included in the study

	Lean dogs	Overweight dogs
	n = 12	n = 16
	BCS (4–5)	BCS (6–8)
Frequency of scraps, treats and chews^a^	6 (4.5–7)	6 (3.5–7)
*Pooled scores, median (interquartile range)*		
Daily energy intake from commercial diet^b^	2:10	2:14
*n*_*75* *%*_ *:n*_*100* *%*_		
Wet or dry commercial diet^c^	1:11	0:16
*n*_wet_:*n*_dry_		
Main protein source in commercial diet^d^	8:1:1:1:1:0	10:4:1:0:0:1
*n*_C_:*n*_BP_:*n*_B_:*n*_CT_:*n*_S_:*n*_WD_		
Calorie restricted commercial diet^e^	2:10	5:11
*n*_yes_:*n*_no_		
Main macronutrient source of total ME^f^	3:9	4:12
*n*_F_:*n*_NFE_		
l-carnitine additives^g^	3:9	2:14
*n*_yes_:*n*_no_		

Summary of the background diet received by dogs in their home environment. Body condition score (BCS) was clinically evaluated by the same veterinarian (JS). P < 0.05 was considered significant in all analyses. NS, non-significant

^a^The frequencies with which dogs were given table scraps and rewarded with training treats and dog chews during 2 weeks preceding the study were evaluated from daily food diaries provided by the dog owners. Scraps, treats and chews were scored separately as follows: 0 (never), 1 (once per 2 weeks), 2 (1–3 times per week), 3 (daily). Scores for scraps, treats and chews were then pooled for each dog and the medians and (interquartile ranges) for lean and overweight groups were calculated. The difference in total scores between body condition groups was analysed by The Mann–Whitney U test (NS)

^b^The proportion (%) of total daily energy intake coming from a complete commercial diet in home environment was estimated by the dog owner. Group differences analysed by Fisher´s exact test (NS)

^c^Number of dogs fed wet or dry complete commercial diet in the home environment. All dry diets were heat treated while the wet diet received by one dog was a frozen raw formula. Group differences analysed by Fisher´s exact test (NS)

^d^Number of dogs fed a main protein source (in complete commercial diet) from: *C* Chicken, *BP* Beef and Pork, *B* Beef, *CT* Chicken and Turkey, *S* Salmon, *WD* Wisent and Deer. Group differences analysed by Chi square test for trend (NS)

^e^Number of dogs fed a calorie-restricted complete commercial diet in home environment (indicated as a *Light diet* according to the manufacturer). Group differences analysed by Fisher´s exact test (NS)

^f^Metabolisable energy (ME) mainly from: *F* Fat (about 40% of total ME), *NFE* Nitrogen free extract (about 40–65% of total ME). No dog had proteins as the main macronutrient source of total ME. Group differences analysed by Fisher´s exact test (NS)

^g^Number of dogs fed a complete commercial diet containing l-carnitine supplementation (according to the manufacturer) in home environment. Group differences analysed by Fisher´s exact test (NS)

### Assessment of health status and body condition

Each dog underwent a standard physical examination (assessment of general condition, skin condition, rectal temperature, visible mucus membranes, palpable lymph nodes, heart and lung auscultation, abdominal palpation and gait). The dogs were weighed and photographed. Routine haematology and serum biochemistry analyses (alanine aminotransferase, alkaline phosphatase, fasting bile acids, creatinine, urea, glucose, fructosamine, total protein, albumin, C-reactive protein, total thyroxine, thyroid stimulating hormone, sodium, potassium and chloride) were performed on fasting blood samples. Urine was analysed by a standard dipstick chemistry test and by refractometry. Some minor health problems (slightly stiff gait and mild lameness, mild periodontitis, palpable peri-articular osteophyte formation and skin furunculosis) were detected in 11 dogs. None of these dogs were excluded, as vital parameters, haematology, serum biochemistry and urine analysis were within reference ranges for healthy dogs.

The dogs were assigned a clinical body condition score (BCS) according to a 9-point scale [32] by the same veterinarian (JS) and the cut-off for overweight (BCS ≥ 6) as suggested by the scoring scale was applied to categorize dogs as lean or overweight. Based on BCS, the lean group (BCS 4–5) consisted of 12 dogs (mean ± SD, age 5.3 ± 1.4 years, body weight 34.8 ± 2.5 kg) and the overweight (BCS 6–8) group consisted of 16 dogs (age 5.2 ± 1.6 years, body weight 39.5 ± 4.6 kg), where calculated lean body weight was 36.2 ± 3.3 kg. Body weight differed significantly between body condition groups (P = 0.004), while age and ideal body weight did not. The frequencies, with which the dogs were awarded table scraps, treats or dog chews did not differ between body condition groups (Table 1). Serum leptin concentration was used to verify the clinical body condition grouping [30].

### Meal-challenge test

For the meal challenge, all dogs were given half their daily energy requirement (DER) as a high-fat mixed-meal. The equation used to compute DER (131 kcal × body weight _kg_^0.75^) was a prediction equation from existing data on adult Labrador Retriever dogs [33]. In the DER equation, the actual body weight of lean dogs and the calculated lean body weight of overweight dogs (body weight minus the weight of the estimated average fat content according to BCS and sex) was used according to a previously constructed calculation based on the 1–9 BCS-scale [12, 32]. The amount test feed in grams received by lean dogs (mean ± SD, 222 ± 12 g) and overweight dogs (228 ± 16 g) did not differ between groups. The test meal was weighed and served with water added (same amount in grams as the individual test meals). The test feed (Science Plan™ Canine Adult Performance, Hills, Etten Leur, the Netherlands) provided 4230 kcal/kg, with 51% of the metabolisable energy as fat, 26% as carbohydrate and 23% as protein. Taurine, omega-3 and omega-6 fatty acids, beta-carotene and vitamins A, C, D and E were added (composition of the macronutrients and other dietary components presented as-fed according to the manufacturer). Macronutrient composition and calculated metabolisable energy (as-fed) of the batch of test feed were confirmed by an independent authorised laboratory (Additional file 1). The postprandial period started at the first bite and all 28 dogs voluntarily consumed all food and water within 10 min of being offered. The dogs were given nothing further to eat or drink and were kept indoors until completion of the postprandial samplings.

### Blood sample collections

After the physical examination, a catheter (Venflon ™ Pro 1.1*32 mm, Becton–Dickinson, Singapore, Malaysia) was placed in the distal cephalic vein and blood samples were collected 15 min before (fasting), and then hourly for 4 h after the test meal (postprandial period). The catheter was flushed with 2 mL physiological saline solution after each blood collection. Serum tubes (Hettich Vacuette Z Serum Clot Activator, Greiner bio-one, Kremsmünster, Austria) were left to clot at room temperature for 30 min and then centrifuged at 1500×*g* for 10 min. Plasma tubes (Hettich Vacuette Lithium Heparin, Greiner bio-one, Kremsmünster, Austria) were centrifuged (as above) directly after sampling. Serum and plasma were transferred into polypropylene tubes (SC Micro Tube PCR-PT, Sarstedt AG & Co, Nümbrecht, Germany) and immediately frozen at − 70 °C. Fasting serum was analysed for biochemistry as a predictor of general health status and plasma from all time points were used for NMR analyses. The catheter was removed after the final blood sampling.

### Targeted NMR-based metabolomics analysis

#### Sample preparation, NMR data acquisition and spectral processing

NMR-based metabolomics analysis of plasma samples was performed for samples taken at fasting and postprandial time points. Sample preparation using ultrafiltration [34] was performed with slight modifications. Filter membranes (Nanosep centrifugal filters, 3-kDa cutoff, Pall Life Science, Port Washington, NY, USA) were washed 8 times (0.5 mL water at 4000×*g*, 36 °C) to remove glycerol and 400 µL of each plasma sample were thereafter filtered (10,000×*g*, 4 °C) to remove plasma proteins. Phosphate buffer (150 µL, 0.4 M, pH 7.0), internal TSP standard (30 µL, 20 mM), D_2_O (45 µL) and H_2_O (65 µL) were added to 310 µL filtrate and water to a total sample volume of 600 µL. The sample was transferred to a 5-mm NMR tube. An experimental ^1^H-NMR spectrum was acquired for each prepared sample. All analyses were performed on a Bruker Avance II 600 MHz spectrometer equipped with a cryoprobe and a SampleJet sample changer with a sample cooling system (Bruker Biospin AG, Fällanden, Switzerland) and were run in one batch. For data acquisition, the software TopSpin 3.1 from Bruker was used. Each spectrum was recorded using a zgesgp pulse sequence (Bruker Spectrospin Ltd.) with a relaxation delay of 4 s and 128 scans for each experiment. Measurements were made at 25 °C. The water signal was suppressed by excitation sculpting using arbitrary waveforms and pulse field gradients. All acquired spectra were processed manually in ChenomX (Version 7.5, ChenomX Inc., Canada) and started with phase correction. The internal TSP signal line width was adjusted to obtain a full-width half-maximum value of 1.10 Hz in each experimental spectrum. This was achieved by applying a line broadening factor ≥ 0.3 Hz. The line width adjustments allowed the use of signal heights [35] for later metabolite quantifications.

#### Identification and quantification of plasma metabolites

Metabolite identifications were made using signal pattern recognitions. The observed signals in the experimental spectra were identified based on reported values for chemical shifts, coupling constants and multiplicities, as available in public (the Human Metabolome Database, http://www.hmdb.ca) and commercially available metabolite databases (the metabolite library in ChenomX). A set of 55 metabolites was selected for quantification based on a human protocol [36]. The concentrations of these metabolites were determined in all experimental spectra using a previously described slightly modified strategy for automated quantification algorithm (AQuA) [36] that accounts for complex overlap of experimentally observed signals. The automated quantification algorithm (AQuA) was modified for the experimental spectra from the plasma samples in the current study. As required, a metabolite library was first created using the ChenomX software. The linewidths and positions of the library signals were adjusted to be in agreement with the experimentally observed signals. The metabolite library and experimental spectra were then deployed in the general AQuA workflow, i.e. selection of target signals were used for quantifications, data reduction and AQuA computations. This resulted in estimated sample concentrations (µM) for the 55 metabolites in all experimental spectra. Final plasma concentrations were obtained after accounting for sample dilution (310 µL filtrate to a total sample volume of 600 µL) (Additional file 2).

Each individual metabolite was evaluated using a set of quality indicators [36] to determine whether it was suitable for inclusion in statistics or not. The inclusion criteria for the plasma metabolites quantified by AQuA in the current study were: CV ≤ 20%, metabolite occurrence ≥ 50% of all samples, and no target signal positional deviation between the experimental spectra. It proved possible to quantify 43 of the 55 metabolites with acceptable quality using AQuA. Glucose and lactic acid were excluded from the dataset during statistical evaluations as their large variability suppressed the variation within the other metabolites in the multivariate statistical models. Therefore, 41 metabolites remained and were included in the final multivariate models and univariate statistical analyses (Additional file 3).

### Statistical analysis

Various multivariate models were used as a starting point for identification of discriminant metabolites that varied with time in the meal-challenge test or were related to overweight. The discriminant metabolites were further investigated by mixed model repeated measures analyses. A value of P < 0.05 was considered significant unless otherwise indicated.

#### Multivariate data analysis

Multivariate comparisons were performed on datasets containing plasma metabolite concentrations (n = 41 as x-variables) using commercially available software (SIMCA-P + 13.0 Umetrics, Umeå, Sweden). Randomisation of raw data, pareto-scaling, step-wise removal of up to five outliers in each comparison was applied. Gaussian distribution of the whole dataset within the models was then tested by normal probability plotting. Principal component analysis (PCA) was used to visualise any unconstrained clustering of fasting and each postprandial time point (including all 28 dogs) in four separate models (fasting versus 1, 2, 3, and 4 h respectively). Clustering of lean and overweight dogs within each time point (fasting, 1, 2, 3 and 4 h respectively) was investigated in five separate PCA models. The observed clustering was tested for significance with constrained methods in partial least-squares discriminant analysis (PLS-DA) and the significance of PLS-DA models was tested using cross-validated analysis of variance (CV-ANOVA) [37]. Variable importance of projection (VIP) based on the PLS-DA models was used to determine the most important discriminative metabolites in each significant comparison. Metabolites with VIP > 1 and for which the corresponding jackknife-based 95% confidence intervals (CI) were not close to or included zero were considered discriminative and significant for the observed separations.

#### Stepwise logistic regression analysis

For each time point, binary (i.e. lean and overweight groups of dogs) stepwise logistic regression [38] was used to identify metabolites that were related to overweight (BSC ≥ 6). The Logistic procedure in the SAS package (2014, 9.4 Institute Inc., Cary, NC) was used for this purpose.

#### Mixed model repeated measures analysis

Plasma responses to the meal-challenge test were evaluated by mixed model repeated measures analysis [39] in SAS [38, 40]. In the statistical model, body condition group was defined as an independent variable and the fasting value was included as a time point. The model analysed the response over time from fasting to 4 h after feeding and differences between the lean and overweight groups. Thus, the model was capable of overall and pair-wise comparisons and was able to correct for multiple comparisons within the model by Tukey–Kramer adjustment. Logarithmic transformation of raw data was performed to correct for non-normality (based on the distribution of residuals within the model) in the statistical analysis of carnitine and 3-hydroxybutyrate concentrations. Only discriminant metabolites identified by multivariate models were tested in the mixed model repeated measures analysis and thereafter Bonferroni correction for multiple comparisons was applied. When selection of important metabolites was not preceded by multivariate approaches but instead by physiological importance (i.e. the ketone bodies), Bonferroni correction for multiple comparisons was applied upon the total number of metabolites (level of significance α < 0.0012, 0.05/n = 41).

## Results

### Comparisons of fasting and postprandial plasma metabolomes

Plasma metabolomes from all 28 dogs analysed by multivariate PCA models had clear visual separations between the respective postprandial time points (1, 2, 3 and 4 h) and the fasting time point (Additional file 4). The significant separations between fasting and respective postprandial time points were confirmed by analyses in PLS-DA models and by CV-ANOVA (Table 2). The VIP analyses based on the PLS-DA models identified 11 discriminative metabolites (Table 2), of which 10 were found to be different among time points in the mixed model repeated measures analyses (Bonferroni correction for multiple comparisons was applied, level of significance α < 0.005, 0.05/n = 11) (Table 3). The discriminative metabolites were all amino acids and showed increasing concentrations in response to the meal challenge in all cases except for glutamine, which decreased (Table 3). Although not discriminant between time points in the multivariate analysis, the two organic acids 3-hydroxybutyrate and acetoacetate were found to be significantly different over time in the mixed model repeated measures analysis (P < 0.0001 for both) demonstrating a postprandial decrease in 3-hydroxybutyrate and increase in acetoacetate (Additional file 5).

**Table 2:** Plasma metabolites differentiating fasting and postprandial metabolomes in multivariate discriminant analysis, with all 28 dogs included

Metabolite	Fasting vs 1h^a^ VIP (CI)	Fasting vs 2h^b^ VIP (CI)	Fasting vs 3h^c^ VIP (CI)	Fasting vs 4h^d^ VIP (CI)
Alanine	2.7 (1.5)	2.5 (0.8)	2.2 (1.4)	1.8 (0.5)
Arginine	1.6 (0.5)	1.3 (0.6)	1.1 (0.4)	NS
Glutamine	NS	NS	1.4 (1.2)	1.6 (1.1)
Glycine	2.4 (1.2)	2.0 (0.5)	1.6 (1.0)	1.2 (0.6)
Isoleucine	1.2 (0.5)	1.5 (0.3)	1.6 (0.4)	1.8 (0.3)
Leucine	1.4 (0.6)	1.9 (0.4)	2.1 (0.4)	2.3 (0.4)
Lysine	1.1 (0.8)	NS	NS	NS
Ornithine	1.1 (0.4)	1.1 (0.3)	1.0 (0.3)	NS
Proline	3.1 (0.6)	3.1 (0.3)	2.9 (0.6)	2.9 (0.5)
Threonine	1.2 (1.1)	NS	1.1 (1.0)	NS
Valine	1.7 (0.8)	2.3 (0.4)	2.8 (0.6)	3.1 (0.5)

Principal least-squares discriminant analyses using the different time points (fasting versus 1–4 h postprandial, respectively) as predefined groups. All fitted models showed significant separations a–d (presented below). *VIP* Variable importance of projection, *CI* confidence interval, *NS* not significant

(R^2^Y = Percent of variation in data set explained by model, a measure of fitness)

(Q^2^Y = Percent of variation in data set predicted by model)

^a^One component fitted: R^2^Y = 0.39, Q^2^Y = 0.31; cross-validated ANOVA: P = 0.00014

^b^One component fitted: R^2^Y = 0.55, Q^2^Y = 0.49; cross-validated ANOVA: P = 0.00000004

^c^One component fitted: R^2^Y = 0.64, Q^2^Y = 0.58; cross-validated ANOVA: P = 0.0000000005

^d^Two components fitted: R^2^Y = 0.71, Q^2^Y = 0.63; R^2^Y = 0.11, Q^2^Y = 0.09; cross-validated ANOVA: P = 0.0000000002

**Table 3:** Discriminant plasma metabolites differentiating fasting and postprandial metabolomes significant in mixed model repeated measures analysis, all the included 28 dogs

Metabolite	Fasting	Postprandial			SE^b^
	Mean ± SD (µM)	Hours	Mean ± SD (µM)	*P* value^a^	
Alanine	221 ± 50.8	1	249 ± 50.6	< 0.0001	5.94
		2	265 ± 48.7	< 0.0001	7.97
		3	264 ± 44.4	< 0.0001	9.27
		4	241 ± 44.5	0.27	10.2
Arginine	94.1 ± 10.6	1	104 ± 14.0	< 0.0001	1.91
		2	106 ± 15.8	< 0.0001	2.51
		3	106 ± 16.1	0.0006	2.88
		4	101 ± 13.2	0.16	3.12
Glutamine	431 ± 67.5	1	414 ± 49.5	0.04	5.68
		2	396 ± 50.2	0.0002	7.75
		3	392 ± 42.4	0.0005	9.16
		4	389 ± 48.1	0.0007	10.2
Glycine	149 ± 22.7	1	173 ± 27.6	< 0.0001	3.74
		2	178 ± 33.5	< 0.0001	5.02
		3	177 ± 40.2	< 0.0001	5.85
		4	166 ± 32.2	0.06	6.43
Isoleucine	35.3 ± 6.1	1	40.8 ± 5.4	< 0.0001	1.01
		2	46.6 ± 6.9	< 0.0001	1.31
		3	51.1 ± 6.7	< 0.0001	1.47
		4	52.0 ± 6.8	< 0.0001	1.57
Leucine	64.6 ± 9.9	1	73.1 ± 8.9	< 0.0001	1.70
		2	83.5 ± 11.6	< 0.0001	2.18
		3	90.8 ± 10.6	< 0.0001	2.44
		4	92.4 ± 10.7	< 0.0001	2.59
Lysine	82.0 ± 20.0	1	88.3 ± 18.2	0.002	1.88
		2	87.9 ± 18.1	0.12	2.56
		3	83.8 ± 15.9	0.97	3.02
		4	76.2 ± 15.3	0.47	3.37
Ornithine	8.2 ± 3.3	1	11.9 ± 2.3	< 0.0001	0.48
		2	14.7 ± 3.9	< 0.0001	0.64
		3	15.2 ± 4.4	< 0.0001	0.74
		4	14.1 ± 4.1	< 0.0001	0.81
Proline	111 ± 17.6	1	139 ± 20.8	< 0.0001	3.63
		2	157 ± 27.3	< 0.0001	4.71
		3	163 ± 29.0	< 0.0001	5.32
		4	157 ± 25.5	< 0.0001	5.70
Valine	105 ± 14.7	1	117 ± 13.6	< 0.0001	2.41
		2	133 ± 16.6	< 0.0001	3.13
		3	148 ± 16.3	< 0.0001	3.53
		4	153 ± 16.2	< 0.0001	3.78

Mixed model repeated measures analysis with significance level P < 0.05 and Tukey–Kramer adjustment within model. Fasting and postprandial metabolite concentrations (µM) are shown as mean ± standard deviation (SD). Ten metabolites were overall different between time points (glutamine P = 0.0004 the remaining nine P < 0.0001)

^a^P-values for comparisons between fasting and each postprandial time point, respectively

^b^Standard error (SE) for comparisons between fasting and postprandial time points

### Comparisons of plasma metabolomes in lean and overweight dogs

**Fig. 1:**
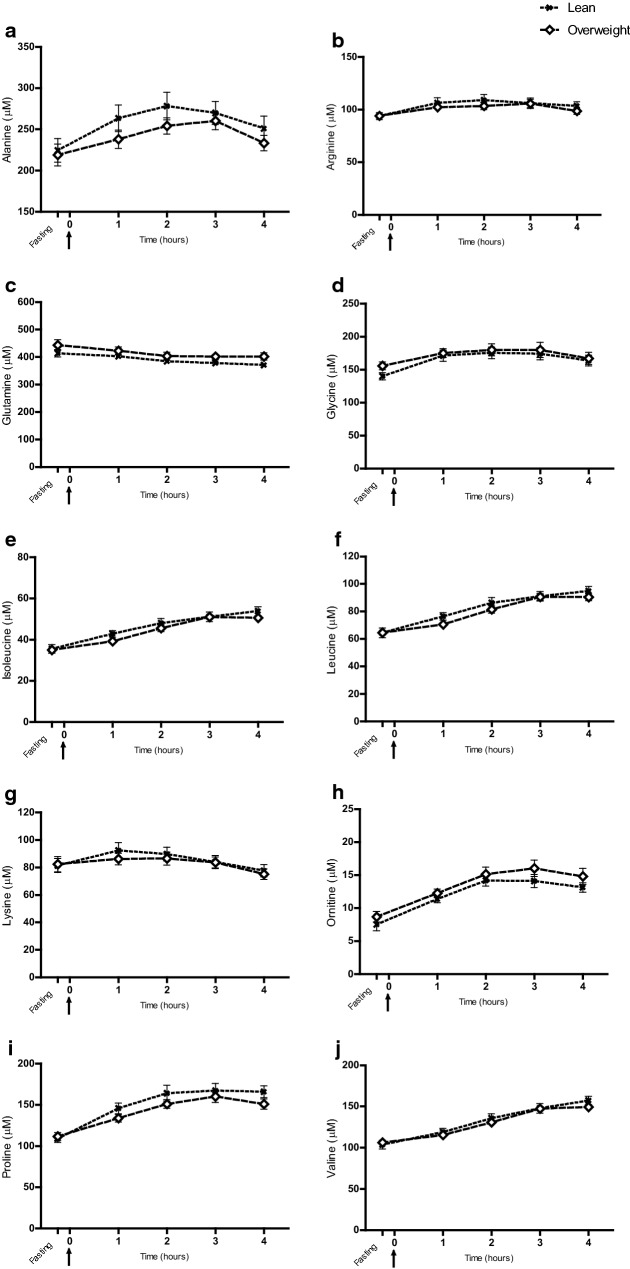
Discriminant metabolites significant over time in the meal-challenge test, analysed with respect to body condition groups. Dogs were divided into two body condition groups: lean (BCS 4–5, n = 12) and overweight (BCS 6–8, n = 16), and the mixed model repeated measures analysis was applied. Values given as µM concentrations (mean ± SEM). Fasting plasma samples were taken 15 min before serving of a test meal at time 0 (arrow) and metabolite concentrations in lean and overweight dogs are shown as response curves from fasting to 4 h after feeding. No time-dependent variations in overall metabolite response with regards to body condition groups were found (**a**–**j**)

**Fig. 2:**
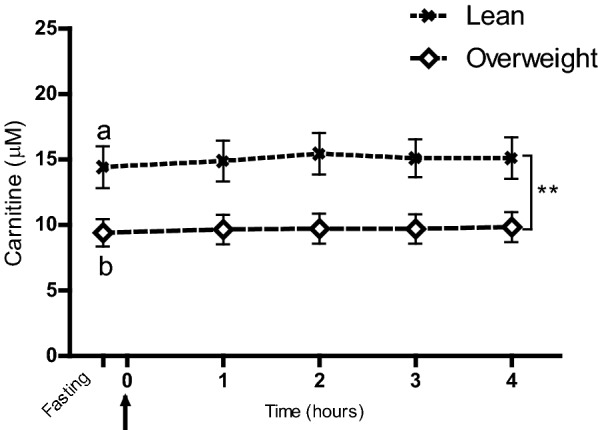
Differences in plasma carnitine concentrations between lean and overweight dogs during the meal-challenge test. Dogs were divided into two body condition groups: lean (BCS 4–5, n = 12) and overweight (BCS 6–8, n = 16), and the mixed model repeated measures analysis was applied. Values are given as µM concentrations (mean ± SEM). Fasting plasma samples were taken 15 min before serving of a test meal at time 0 (arrow) and carnitine concentrations in lean and overweight dogs are shown as response curves from fasting to 4 h after feeding. Significant differences in overall responses between body condition groups (**P < 0.01) are indicated and different letters (a and b) indicate significant differences between body condition groups within the fasting time point (P < 0.05)

No separations were visually observed between lean and overweight dogs in the multivariate PCA models, either at fasting or at the 1 to 4 h postprandial time points. This was confirmed by the finding that none of the PLS-DA models could be fitted using lean and overweight dogs as predefined groups at any time point. The 10 amino acids that were discriminant over time in the meal-challenge test did not differ between body condition groups (Fig. 1), nor did the two ketone bodies (Additional file 5). In logistic regression analyses carnitine was shown to be related to overweight at all time points (P ≤ 0.03 for all), while no other metabolites were identified by this statistical model. In the mixed model repeated measures analysis, carnitine was found to be overall lower in overweight compared to lean dogs during the whole meal-challenge test (P = 0.005), but the carnitine response was not affected by food intake in either of the groups (Fig. 2). Overweight dogs exhibited a mean carnitine concentration of 9.4 ± 4.2 µM at fasting (about two-thirds of the concentration of lean dogs) and the two body condition groups differed significantly at fasting (P = 0.008) (Fig. 2).

## Discussion

In this study, postprandial plasma metabolomes were identified and quantified to further the understanding of canine postprandial metabolism and to investigate potential metabolic variations related to spontaneous overweightness in healthy dogs. Using an NMR analytical platform and the AQuA quantification technique, a postprandial amino acid response was detected in the canine metabolome and free carnitine was found to be lower in overweight compared to lean dogs.

The measured mean carnitine concentration in overweight dogs (9.4 ± 4.2 µM) was at the very lower range of a proposed reference interval (8–36 µM) for normal fasting plasma carnitine concentrations in dogs [41]. This is interesting as a lower carnitine concentration might reduce lipid metabolism, as carnitine is essential for fatty-acid oxidation. Carnitine also provides a buffer for acyl groups by the reversible formation of acylcarnitines, i.e. carnitine bound to fatty acids. Hence, the lower free carnitine concentration in overweight dogs could possibly be related to adiposity and free carnitine might become depleted due to excessive acylcarnitine formation and buffering in overweight dogs [42]. However, due to the present study design, evaluation of whether the lower carnitine concentrations were a cause or a consequence of weight gain was not possible. Few studies have reported plasma carnitine concentrations in lean and overweight dogs [20, 43, 44]. In these studies, a limited number of laboratory dogs of different sexes and neutering status underwent experimental weight reduction or weight gain and the results did not demonstrate lower free carnitine concentrations in overweight dogs. However, Xu et al. [44] used a highly precise and accurate LC–MS/MS tandem approach which showed plasma carnitine concentrations comparable (although not lower in obese dogs) to the carnitine concentrations measured in the present study, which strengthens the validity of the results obtained by the NMR analysis. One of the studies found that several acylcarnitines (but not free carnitine) were increased during acute weight gain [20]. The reason for discrepancies between study results is not known, but a possible explanation is that carnitine metabolism differs in acute experimental versus spontaneous overweight in dogs. Furthermore, carnitine evaluation is hampered by many different factors such as tissue distribution, fraction (i.e. free carnitine or bound to fatty acids as acylcarnitines), diet, age and time since last meal [15, 41, 42, 45]. In the present study, all dogs were of the same breed and sex and mean age did not differ between body condition groups, suggesting that these factors should not have biased the results. The propensity of some large and medium sized dog breeds to develop carnitine and/or taurine insufficiencies has been proposed [41, 46, 47] and is related to development of dilated cardiomyopathy. In the Swedish dog population, the Labrador Retriever dog is not considered a high risk breed for development of dilated cardiomyopathy [48]. To gain further knowledge about the importance of breed size in relation to overweight with regards to plasma carnitine status, dogs from breeds of different sizes, including groups with different BCSs, should preferably be tested by the same methodology as used in the present study. Although the vast majority of the total body content of carnitine resides in muscle tissues, it has been proposed that plasma carnitine might act as an indicator of carnitine insufficiency at concentrations near the lower limit of the normal reference range [41], as found in the overweight dogs in this study. Since acylcarnitines and tissue distribution of carnitine were not measured by the methods employed in the present study, further research is needed to evaluate these two parameters in relation to spontaneous overweightness in dogs.

In dogs, carnitine is obtained from dietary protein or is endogenously synthesised in the liver from the amino acid lysine and requires the sulphur-amino acid methionine for methylation [49]. One sulphur-amino acid (methionine) was quantified in the present study (Additional file 6), and did not differ between lean and overweight dogs. In people, carnitine concentrations have been shown to decrease by long term vegetarianism [50] and in obesity, carnitine demands have been proposed to increase due to higher metabolic stress [51]. A long term high-fat diet in obese rodents decreased the activity of carnitine biosynthetic genes [52]. It is therefore possible that plasma carnitine concentrations might be affected by type and amount of food intake. Although complete commercial dog foods might vary in concentration of available free carnitine [53], healthy dogs fed solely complete diets [44, 53] were found to be well within recommended reference ranges [41, 54] for plasma free carnitine at fasting. As all dogs in the present study were fed complete diets based on animal proteins, it is therefore unlikely that they should have suffered from diet-related carnitine insufficiency. The fat content and exact amount of given table scraps were not possible to assess, and differences in total fat intake between groups can therefore not be excluded. As the fat content of the diet might influence plasma carnitine concentration in people [55] and rodents [52], possible effects of high or low-fat diets in dogs need further investigation. Long-term intake of diets supplemented with l-carnitine could theoretically reduce endogenous carnitine synthesis, resulting in lower plasma carnitine concentrations. However, only three lean and two overweight dogs were fed complete diets containing l-carnitine additives, suggesting that carnitine supplementation did not interfere vastly with the differences found in mean carnitine concentrations between body condition groups in this study. Altogether, we suggest that the spontaneous adiposity of the overweight dogs probably had a greater impact on carnitine metabolism than possible dietary differences alone.

Interestingly, we found no effect of meal intake on plasma concentration of carnitine although the test food contained meat-derived carnitine precursors (but no l-carnitine additives). Our results thereby indicate that sampling after an overnight fast compared with 1–4 h after a mixed meat-containing meal might generate comparable results on plasma free carnitine concentrations in dogs. However, the impact of recent food intake and different types of foods in the regular diet need to be further studied. Our postprandial results are partly supported by those of a study in people showing that plasma free carnitine might have a delayed postprandial increase, measured at 24 h after a prolonged fast [56]. It is therefore possible that a longer fasting period or a sampling time points greater than 4 h postprandially would have generated different results.

Analyses of postprandial plasma responses in all dogs demonstrated increased concentrations of 9 amino acids. This finding was likely a reflection of amino acids supplied by the test diet as have been shown in humans [27] and to a lesser extent, endogenously synthesised amino acids at the later sampling time points. The sampling scheme detected the peak concentration for 10 out of 11 of the discriminant metabolites, suggesting that the 4-h postprandial sampling period was long enough in relation to the diet composition [28] although return to basal amino acid concentrations would have been ideal. Future meal-challenge studies in dogs should aim for a more frequent sampling scheme in the early postprandial period as well as a longer postprandial period as a whole; however, this was not possible in the present study due to practical reasons and privately owned dogs. All of the discriminative amino acids showed increasing concentrations in response to the meal challenge with the exception of glutamine, the concentration of which decreased. Glutamine is the most abundant free amino acid in the circulation and an important substrate for gluconeogenesis [57, 58], thus, the utilization of glutamine in the fasted state could be pronounced. The decline in glutamine concentrations after feeding was therefore somehow unexpected, as the gluconeogenesis presumably would have been switched off by meal intake. Glutamine supplementation has been shown to increase postprandial fat oxidation in people [59] and the glutamine decrease might thus be related to the metabolism of the high-fat meal. Similar to glutamine, the ketone body 3-hydroxybutyrate decreased in response to the meal challenge, which might represent decreased beta-oxidation of stored body fat and a switch from catabolic to anabolic states. This is a physiological response after food intake and a similar pattern has been found in an NMR-based metabolism study in people [29]. Acetoacetate, which is the primary ketone body formed, showed continuously increasing concentrations from fasting to the 4-h postprandial time point. The concentration of this metabolite commonly decreases after food intake, along with rising insulin concentrations. However, as formation of acetoacetate is also stimulated by high fat intake [60], it is possible that the high-fat content of the test meal had an impact on the postprandial plasma acetoacetate response in this study.

A postprandial response in branched-chain amino acids related to food intake was detected in our data, but, in contrast to findings in overweight people [18, 19], the overweight group did not exhibit higher concentrations compared with the lean group. In fact, no time-dependent variations with regards to overweight dogs were found in the meal-challenge test. Interestingly, acutely overfed dogs in a previous study [20] showed a biphasic pattern of fasting branched-chain amino acid concentrations being initially elevated, but subsequently returning to basal concentrations in more chronic obesity, the latter stage somehow comparable to the results of our study. All spontaneously overweight dogs investigated in our study were intact males, were reported to have had a stable weight before sampling and no experimental overfeeding was conducted. In addition, none of the dogs were obese or profoundly insulin-resistant [13] as could be expected in experimental acute weight gain. Obese dogs often become insulin-resistant, but seldom become hyperglycaemic or develop type 2 diabetes [12, 61] in the same way as humans do [62]. All these parameters could potentially explain differences in the patterns of branched-chain amino acids found between dogs and humans and between chronic and acute models of canine overweight.

The diet in home environment was not modified in order to avoid the risk of changing the body weight preceding sample collection. Owner-feeding habits were investigated by food diaries and no statistical differences were found between lean and overweight dogs in the frequencies with which dogs were given other feeds than complete diets. The possibility of small differences in the background diet that were not detected by the food diaries is a potential study limitation. However, one study in people [25] found that the postprandial plasma metabolite response was stable over time and largely independent of background diet. Therefore, potential differences in the background diet probably had a minor impact on postprandial study results in the meal-challenge test. Despite great efforts, a relatively low number of dogs were included in the present study. Hence, negative findings cannot be absolutely trusted in this study, and in future studies, power calculations should be performed to assess the adequate sample sizes needed. However, the multivariate models used in the present study all proved to be robust and were able to detect responses over time as well as group differences in one metabolite. It would have been desirable to include more obese (BCS 8–9) but yet healthy dogs, but such dogs proved extremely difficult to enrol. An objective confirmation of the clinical BCS (e.g. dual energy X-ray absorptiometry) might have strengthened the BCS results, but this method was not available at the time of the study. The fact that dogs from only one breed and sex were used in this study has presumably reduced the individual variation and made it possible to detect subtle metabolic changes despite a relatively small sample size. However, studies on larger cohorts of various breeds and with different background feeding regimens are needed before results are extrapolated to the dog population as a whole.

## Conclusions

This study showed that a metabolomics approach can enhance the understanding of canine postprandial metabolism. A postprandial amino acid response was detected, but no time-dependent metabolic variations with regards to overweight were found. Plasma free carnitine was identified as a metabolite related to overweight in this cohort of healthy Labrador Retriever dogs and the lower concentration could indicate a potential carnitine insufficiency linked to spontaneous adiposity and altered lipid metabolism.

## Additional files

**Additional file 1.** Test-feed analysis, as fed.

**Additional file 2.** Plasma metabolite concentrations (µM) of the 55 metabolites quantified with the automated quantification algorithm (AQuA).

**Additional file 3.** Evaluation of individual target metabolites quantified with the automated quantification algorithm (AQuA).

**Additional file 4.** Principal component analysis score plots showing fasting and the 1–4 h postprandial metabolomes.

**Additional file 5.** Plasma ketone bodies in the meal-challenge test significant over time in the mixed model repeated measures analysis.

**Additional file 6.** The sulfur amino acid methionine analysed by the mixed model repeated measures in the meal-challenge test.

## Abbreviations

AQuA: automated quantification algorithm
BCS: body condition score
CI: confidence interval
CV-ANOVA: cross-validated analysis of variance
DER: daily energy requirements
NMR: nuclear magnetic resonance
PCA: principal component analysis
PLS-DA: partial least-squares discriminant analysis
SD: standard deviation
VIP: variable importance of projection
