# Supplementary material for: Plasma metabolomics reveals lower carnitine concentrations in overweight Labrador Retriever dogs
Source: Acta Vet Scand. 2019 Feb 26;61:10. doi: 10.1186/s13028-019-0446-4 (PMC6390349; doi:10.1186/s13028-019-0446-4)
Supplement: Supplementary file 1 — Additional file 1. Test-feed analysis, as fed. [file 13028_2019_446_MOESM1_ESM.pdf]

**Additional file 1.** Test-feed analysis, as fed [a]

| **Analysis** | **Results** (deviation) | **Method/Reference** | **Lab** |
|---|---|---|---|
| LW03P Ash | 5.89 g/100 g (± 10%) | 2009/152/EU modified | EUSELI |
| LP022 Crude protein | 28.2 g/100 g (± 7%) | Dumas (Nx6.25) | EUSELI |
| LP00W Alanine | 17.3 g/kg (± 8%) | SS-EN ISO 13903:2005 | EUSELI |
| LP00W Arginine | 19.4 g/kg (± 8%) | SS-EN ISO 13903:2005 | EUSELI |
| LP00WAsparagine and Asparagine acid | 22.3 g/kg (± 8%) | SS-EN ISO 13903:2005 | EUSELI |
| LP00W Cystine | 7.3 g/kg (± 8%) | SS-EN ISO 13903:2005 | EUSELI |
| LP00W Fenylalanine | 12.6 g/kg (± 8%) | SS-EN ISO 13903:2005 | EUSELI |
| LP00W Glutamine acid | 38.1 g/kg (± 8%) | SS-EN ISO 13903:2005 | EUSELI |
| LP00W Glycine | 24.1 g/kg (± 8%) | SS-EN ISO 13903:2005 | EUSELI |
| LP00W Histidine | 5.1 g/kg (± 8 %) | SS-EN ISO 13903:2005 | EUSELI |
| LP00W Hydroxiproline | 6.0 g/kg (± 8%) | SS-EN ISO 13903:2005 | EUSELI |
| LP00W Isoleucine | 13.1 g/kg (± 8%) | SS-EN ISO 13903:2005 | EUSELI |
| LP00W Lysine | 13.6 g/kg (± 8%) | SS-EN ISO 13903:2005 | EUSELI |
| LP00W Metionine | 4.4 g/kg (± 8%) | SS-EN ISO 13903:2005 | EUSELI |
| LP00W Ornitine | 0.3 g/kg (± 8%) | SS-EN ISO 13903:2005 | EUSELI |
| LP00W Proline | 22.1 g/kg (± 8%) | SS-EN ISO 13903:2005 | EUSELI |
| LP00W Serine | 19.3 g/kg (± 8%) | SS-EN ISO 13903:2005 | EUSELI |
| LP00W Threonine | 11.9 g/kg (± 8%) | SS-EN ISO 13903:2005 | EUSELI |
| LP00W Tyrosine | 9.0 g/kg (± 8%) | SS-EN ISO 13903:2005 | EUSELI |
| LP00W Valine | 17.1 g/kg (± 8%) | SS-EN ISO 13903:2005 | EUSELI |
| LP00W Sum amino acids | 286.7 g/kg | SS-EN ISO 13903:2005 | EUSELI |
| LP089 Crude fat | 23.2 g/100 g (± 10%) | EU DIR 98/64 modified | EUSELI |
| LP05C Total dietary fibre | 8.83 g/100 g (± 15%) | AOAC 985.29 | EUSELI |
| LP05M Total sugar | 7.0 g/100 g (± 15%) | Total sugar by Ekelund [b] | EUSELI |
| MJ010 Starch | 24.30 % | In house method [c] | EUNOTR2 |
| LW01M Dry matter | 92.7 g/100 g (± 10%) | 2009/152/EU modified | EUSELI |

[a] Analysis performed by Eurofins Food & Agro Testing Sweden AB, Lidköping, Sweden. www.eurofins.se

[b] Ekelund Sigvard, Statens lantbrukskemiska kontrollanstalt Meddelande 28 (1966), bilaga VIII

[c] Simple sugars not included

Ash (A) from analysis: 5.6 g/100g
Crude protein (CP) from analysis: 28.2 g/100g
Crude fat (CF) from analysis: 23.2 g/100g
Dietary fibre (DF) from analysis: 8.8 g/100g
Nitrogen free extract (NFE) from analysis: Total sugar 7.0 g/100g + Starch 24.3 g/100g = 31.3 g/100g

Calculated NFE from test-feed analysis
NFE: 100 - (A 5.6 + CP 28.2 + CF 23.2 + DF 8.8) = 34.2 g/100 g (as fed)

Calculated metabolisable energy (ME) according to test-feed analysis: 420 kcal/100g (as fed)
(Atwater factors used: CF 8.7, CP 3.5 and NEF 3.5)
28.2*3.5 = 98.7/420 (23.5% of ME as protein)
23.2*8.7 = 201.84/420 (48% of ME as fat)
34.2*3.5 = 119.7/420 (28.5% of ME as calculated NFE from analysis)

31.3*3.5 = 109.6/415 (27% of ME as NFE from analysis) (gives 410 kcal/100g as fed)

ME according to manufacturer: 423 kcal/100g (as fed)
23% of ME as protein
51% of ME as fat
26% of ME as NFE
