# Supplementary material for: Plasma metabolomics reveals lower carnitine concentrations in overweight Labrador Retriever dogs
Source: Acta Vet Scand. 2019 Feb 26;61:10. doi: 10.1186/s13028-019-0446-4 (PMC6390349; doi:10.1186/s13028-019-0446-4)
Supplement: Supplementary file 2 — Additional file 2. Plasma metabolite concentrations (µM) of the 55 metabolites quantified with the automated quantification algorithm (AQuA). [file 13028_2019_446_MOESM2_ESM.pdf]

Additional file 2. Plasma metabolite concentrations (µM) of the 55 metabolites quantified with the automated quantification algorithm (AQuA).

Additional file 2A

Plasma concentrations (µM) of 11 of the in total 55 selected metabolites quantified with AQuA

Sample ID*	1_2_Propane-diol	1_Methylhist-idine	2_Aminobut-yrate	2_Hydroxyb-u-tyrate	2_Hydroxyi-sovalerate	2_Ketogluta-rate	2_Oxoisocap-roate	2_Propanol	3_Hydroxyb-utyrate	3_Methyl_2-oxovalerate	3_Methylhist-idine
01a-Fhr	0.0	0.0	18.9	18.1	0.0	12.7	2.9	2.0	37.5	3.9	0.0
01b-1hr	0.0	0.0	20.0	17.2	0.0	12.4	2.0	0.0	12.6	0.0	0.0
01c-2hr	0.0	0.0	17.6	12.9	2.9	11.5	2.1	0.0	9.9	3.3	0.0
01d-3hr	0.0	0.0	16.3	9.4	0.0	11.4	2.4	1.6	10.1	4.4	0.0
01e-4hr	0.0	0.0	13.5	9.9	0.0	10.2	1.9	1.9	11.6	4.3	0.0
02a-Fhr	0.0	0.0	10.2	9.1	0.0	12.2	2.2	0.0	14.1	3.7	21.0
02b-1hr	0.0	0.0	14.4	11.0	0.0	11.3	2.5	0.0	10.7	0.0	19.2
02c-2hr	0.0	0.0	14.4	13.2	0.0	10.5	0.0	0.0	12.5	0.0	0.0
02d-3hr	0.0	0.0	12.5	11.4	0.0	10.9	0.0	0.0	11.9	0.0	17.5
02e-4hr	0.0	0.0	15.0	11.2	0.0	11.1	1.8	0.0	9.4	0.0	20.2
03a-Fhr	0.0	0.0	14.6	13.8	0.0	13.7	3.1	0.0	26.2	4.7	23.5
03b-1hr	0.0	0.0	14.9	14.1	0.0	12.6	2.5	0.0	13.0	4.0	22.3
03c-2hr	0.0	0.0	16.7	13.6	0.0	12.0	2.7	0.0	11.3	4.0	22.4
03d-3hr	0.0	0.0	17.3	13.2	0.0	11.6	2.8	1.8	12.1	4.4	22.0
03e-4hr	0.0	0.0	14.8	12.8	0.0	11.6	1.9	0.0	11.2	4.2	22.0
05a-Fhr	3.7	0.0	5.6	6.1	0.0	10.1	3.3	0.0	9.6	3.6	15.8
05b-1hr	3.2	0.0	8.2	5.9	0.0	9.9	1.5	0.0	8.7	0.0	14.0
05c-2hr	2.9	0.0	11.0	8.5	0.0	10.9	2.0	0.0	8.1	3.5	14.6
05d-3hr	3.1	0.0	13.1	11.6	0.0	12.9	1.9	0.0	9.2	4.2	24.5
05e-4hr	2.9	0.0	14.9	15.3	0.0	12.2	2.2	1.4	10.1	4.2	25.2
06a-Fhr	0.0	0.0	18.6	20.6	3.6	16.5	4.4	1.8	20.4	6.0	21.6
06b-1hr	0.0	0.0	20.5	20.7	0.0	15.4	4.0	0.0	14.1	5.1	21.5
06c-2hr	0.0	0.0	21.4	15.7	0.0	12.6	4.0	0.0	11.4	4.5	21.9
06d-3hr	0.0	0.0	23.1	14.9	0.0	14.1	4.7	0.0	11.7	5.4	21.2
06e-4hr	0.0	0.0	15.7	11.5	0.0	13.6	3.3	0.0	12.5	6.1	20.7
07a-Fhr	0.0	0.0	16.5	12.6	0.0	16.0	2.9	0.0	24.8	4.6	20.3
07b-1hr	0.0	0.0	16.8	11.2	0.0	13.8	2.6	0.0	11.7	3.7	16.7
07c-2hr	0.0	0.0	18.6	12.2	0.0	15.3	3.1	0.0	8.5	4.6	19.6
07d-3hr	0.0	0.0	15.8	10.9	0.0	13.0	2.5	1.4	9.9	4.3	21.6
07e-4hr	0.0	0.0	15.9	9.6	0.0	14.2	2.3	0.0	9.7	5.1	21.3
08a-Fhr	0.0	0.0	11.8	13.2	0.0	10.2	2.2	0.0	11.0	0.0	17.0
08b-1hr	0.0	0.0	15.1	13.1	0.0	11.1	2.2	0.0	9.1	3.3	15.2
08c-2hr	0.0	0.0	15.3	10.8	0.0	11.3	1.9	1.4	8.0	3.9	19.8
08d-3hr	0.0	0.0	13.8	12.2	0.0	10.6	2.4	0.0	8.6	4.7	15.4
08e-4hr	0.0	0.0	13.4	12.1	0.0	10.7	2.1	0.0	8.3	4.0	21.0
09a-Fhr	24.1	0.0	13.9	13.7	0.0	14.2	2.4	2.2	10.6	3.4	16.8
09b-1hr	19.7	0.0	17.0	12.5	0.0	13.1	1.9	0.0	8.9	3.0	17.8
09c-2hr	17.3	0.0	18.9	14.3	0.0	11.9	2.3	0.0	9.3	3.9	16.7

09d-3hr	13.1	0.0	18.7	13.3	0.0	11.4	2.0	0.0	8.6	3.6	13.0
09e-4hr	10.8	0.0	18.0	15.0	0.0	13.0	2.5	0.0	8.0	3.5	16.4
10a-Fhr	0.0	0.0	12.3	10.5	0.0	9.2	1.9	1.6	7.5	0.0	13.2
10b-1hr	0.0	0.0	14.8	11.6	0.0	9.4	0.0	0.0	7.1	0.0	16.7
10c-2hr	0.0	0.0	15.4	12.5	0.0	10.8	2.1	2.4	7.3	3.7	20.2
10d-3hr	0.0	0.0	18.3	17.3	0.0	12.3	2.4	2.1	9.6	3.8	20.9
10e-4hr	0.0	0.0	17.1	15.4	0.0	10.9	2.1	1.5	8.1	3.5	19.6
11a-Fhr	0.0	0.0	16.5	19.4	0.0	18.5	3.5	1.6	16.4	5.1	25.0
11b-1hr	0.0	0.0	18.7	15.2	0.0	12.9	2.6	0.0	10.8	3.9	23.4
11c-2hr	0.0	0.0	18.0	15.8	0.0	15.8	2.5	0.0	11.5	4.3	24.4
11d-3hr	0.0	0.0	16.2	16.3	0.0	15.0	2.8	1.3	11.6	4.5	22.9
11e-4hr	0.0	0.0	14.8	16.0	0.0	12.2	2.3	0.0	10.9	4.2	0.0
12a-Fhr	0.0	0.0	11.9	9.8	0.0	12.7	2.1	0.0	11.2	3.5	16.4
12b-1hr	0.0	0.0	12.9	11.5	0.0	11.3	0.0	0.0	10.0	0.0	0.0
12c-2hr	0.0	0.0	13.5	9.8	0.0	11.1	1.5	1.3	7.9	3.2	17.2
12d-3hr	0.0	0.0	13.3	11.4	0.0	11.4	0.0	0.0	10.7	3.9	12.8
12e-4hr	0.0	0.0	13.5	10.8	0.0	12.9	1.6	1.8	9.2	3.9	18.4
13a-Fhr	0.0	0.0	13.2	13.3	0.0	11.5	2.4	0.0	8.2	0.0	18.4
13b-1hr	0.0	0.0	16.1	11.4	0.0	10.8	1.8	1.8	7.1	0.0	17.9
13c-2hr	0.0	0.0	13.1	11.4	0.0	8.7	0.0	0.0	8.3	0.0	16.1
13d-3hr	0.0	0.0	13.6	14.5	0.0	12.2	2.2	1.3	8.4	4.1	17.1
13e-4hr	0.0	0.0	14.0	12.1	0.0	8.7	1.5	0.0	6.9	4.3	11.9
14a-Fhr	0.0	0.0	13.3	12.8	0.0	15.6	3.0	0.0	10.0	4.4	15.9
14b-1hr	0.0	0.0	15.5	21.7	0.0	14.1	2.9	0.0	9.7	0.0	18.8
14c-2hr	0.0	0.0	18.9	19.5	0.0	13.6	2.4	1.5	8.2	3.3	17.9
14d-3hr	0.0	0.0	19.4	20.2	0.0	14.2	3.0	2.0	8.5	4.5	19.5
14e-4hr	0.0	0.0	17.4	21.1	0.0	13.8	3.1	2.5	10.0	4.9	0.0
15a-Fhr	3.6	0.0	13.8	16.4	0.0	13.0	2.7	0.0	14.8	4.4	30.4
15b-1hr	3.5	0.0	12.4	13.3	0.0	13.0	3.1	0.0	17.1	3.9	32.2
15c-2hr	3.3	0.0	12.1	13.3	0.0	12.8	2.7	1.4	14.0	4.1	19.1
15d-3hr	3.8	0.0	10.5	12.1	0.0	14.2	3.6	2.4	24.7	5.1	22.4
15e-4hr	0.0	0.0	14.9	17.6	0.0	13.5	2.9	0.0	15.2	4.7	27.6
16a-Fhr	0.0	0.0	14.0	14.8	0.0	17.5	3.4	2.2	6.3	4.0	22.1
16b-1hr	0.0	0.0	18.5	12.9	0.0	15.6	1.9	1.8	8.2	0.0	23.1
16c-2hr	0.0	0.0	22.2	17.9	0.0	19.6	3.0	1.8	9.3	4.8	28.1
16d-3hr	0.0	0.0	19.0	12.4	0.0	16.2	0.0	0.0	7.0	0.0	17.4
16e-4hr	0.0	0.0	18.2	12.3	0.0	16.4	2.1	1.8	6.2	0.0	22.7
17a-Fhr	0.0	0.0	9.7	8.9	0.0	14.8	2.2	2.6	8.4	0.0	16.5
17b-1hr	0.0	0.0	11.9	8.3	0.0	12.3	1.4	2.3	7.0	0.0	21.7
17c-2hr	0.0	0.0	12.8	9.4	0.0	12.8	1.8	2.4	7.1	0.0	20.8
17d-3hr	0.0	0.0	11.7	9.2	0.0	11.7	1.7	2.3	8.2	3.9	25.3
17e-4hr	0.0	0.0	10.0	10.8	0.0	10.6	0.0	3.1	9.2	4.2	14.8
18a-Fhr	2.6	0.0	18.9	16.6	0.0	20.1	3.2	6.2	25.6	4.0	12.5
18b-1hr	2.7	0.0	19.1	13.5	0.0	15.7	2.3	3.0	10.5	3.6	21.1
18c-2hr	0.0	0.0	18.4	14.8	0.0	15.6	2.4	4.4	10.9	4.3	20.9
18d-3hr	0.0	0.0	16.6	16.4	0.0	14.1	2.5	2.6	12.5	4.5	0.0
18e-4hr	2.9	0.0	18.1	19.2	0.0	14.7	2.6	2.4	13.2	4.3	23.4
19a-Fhr	0.0	0.0	8.8	8.2	0.0	12.6	2.7	0.0	8.7	3.8	19.4

19b-1hr	0.0	0.0	12.4	9.1	0.0	10.6	1.6	0.0	6.2	0.0	16.4
19c-2hr	0.0	0.0	15.1	11.7	0.0	12.2	2.3	2.0	8.9	4.4	24.7
19d-3hr	0.0	0.0	16.5	12.8	0.0	13.3	2.2	1.8	7.8	4.2	23.0
19e-4hr	0.0	0.0	17.6	19.2	0.0	13.5	2.8	0.0	8.0	4.1	23.0
20a-Fhr	0.0	0.0	11.1	14.3	0.0	12.8	3.8	2.7	14.5	5.0	15.9
20b-1hr	0.0	0.0	13.4	11.9	0.0	11.2	2.2	5.4	9.6	3.8	0.0
20c-2hr	0.0	0.0	11.4	7.4	0.0	9.4	2.2	2.1	8.1	4.4	16.9
20d-3hr	0.0	0.0	10.9	9.1	0.0	10.3	2.5	1.6	8.1	4.6	17.9
20e-4hr	0.0	0.0	15.4	10.3	0.0	10.2	3.9	0.0	9.1	0.0	0.0
21a-Fhr	0.0	0.0	12.4	11.5	0.0	14.9	2.8	2.3	7.3	4.2	20.1
21b-1hr	0.0	0.0	15.9	13.6	0.0	15.3	2.2	2.1	6.7	3.6	15.4
21c-2hr	0.0	0.0	17.5	15.3	0.0	13.4	2.4	1.5	8.5	4.0	24.0
21d-3hr	0.0	0.0	15.5	19.3	0.0	14.2	1.7	0.0	7.5	4.0	0.0
21e-4hr	0.0	0.0	17.0	22.9	0.0	14.0	2.2	0.0	8.1	3.9	0.0
22a-Fhr	9.8	0.0	20.6	31.8	0.0	17.1	2.8	1.8	25.5	0.0	0.0
22b-1hr	8.2	0.0	26.0	29.4	0.0	16.1	2.6	1.7	19.7	3.6	23.8
22c-2hr	7.1	0.0	26.4	33.7	0.0	18.5	3.1	2.4	21.9	5.1	16.3
22d-3hr	5.8	0.0	23.8	30.0	0.0	16.2	2.6	2.1	19.5	5.1	21.4
22e-4hr	5.2	0.0	22.8	25.3	0.0	17.0	2.6	2.0	21.0	5.6	0.0
23a-Fhr	15.9	0.0	21.0	27.2	0.0	15.9	2.7	1.8	15.3	3.4	18.3
23b-1hr	14.5	0.0	26.4	20.7	0.0	14.7	2.8	1.6	12.7	3.9	18.8
23c-2hr	11.2	0.0	23.0	21.9	0.0	14.3	2.9	1.7	13.1	4.3	13.9
23d-3hr	9.7	0.0	22.9	23.9	0.0	14.8	2.5	1.7	12.5	0.0	18.6
23e-4hr	8.1	0.0	23.3	20.9	0.0	13.5	2.3	2.3	12.4	3.8	18.3
24a-Fhr	0.0	0.0	13.0	12.2	0.0	14.3	3.4	3.3	14.2	4.9	17.5
24b-1hr	0.0	0.0	13.8	12.2	0.0	13.7	2.5	1.7	9.6	3.5	12.8
24c-2hr	0.0	0.0	14.3	9.7	0.0	13.0	2.2	0.0	7.4	0.0	0.0
24d-3hr	0.0	0.0	14.3	10.2	0.0	13.2	2.4	0.0	7.1	4.1	17.9
24e-4hr	0.0	0.0	14.2	11.5	0.0	14.3	2.6	0.0	8.8	4.4	0.0
25a-Fhr	5.3	0.0	11.9	11.9	0.0	14.0	2.3	4.1	14.3	4.0	0.0
25b-1hr	5.6	0.0	13.5	11.1	0.0	12.2	1.8	2.1	11.2	0.0	0.0
25c-2hr	5.8	0.0	12.5	10.2	0.0	12.4	2.0	2.5	10.9	3.9	0.0
25d-3hr	5.7	0.0	11.8	9.8	0.0	12.7	2.2	2.3	9.3	4.6	0.0
25e-4hr	5.4	0.0	10.3	8.9	0.0	12.3	2.3	2.1	12.5	4.5	0.0
26a-Fhr	4.1	0.0	15.9	22.6	0.0	21.1	4.6	2.3	19.1	5.2	21.1
26b-1hr	0.0	0.0	14.8	16.8	0.0	16.0	2.5	1.8	17.2	4.0	22.7
26c-2hr	0.0	9.3	16.5	21.8	0.0	16.9	3.0	2.2	13.4	4.5	16.0
26d-3hr	0.0	0.0	16.0	24.8	0.0	17.0	3.1	2.0	11.3	4.2	23.6
26e-4hr	0.0	9.1	18.3	23.7	3.4	14.5	3.3	2.3	13.3	4.7	27.2
27a-Fhr	0.0	0.0	14.7	14.8	0.0	17.8	2.6	2.9	11.2	4.2	19.1
27b-1hr	0.0	0.0	15.8	19.1	0.0	16.6	2.4	2.0	10.3	3.6	20.3
27c-2hr	0.0	0.0	18.0	20.9	0.0	15.7	1.9	2.3	10.6	3.7	13.4
27d-3hr	0.0	0.0	19.0	24.4	0.0	15.2	3.7	2.5	12.5	4.9	19.1
27e-4hr	0.0	0.0	19.0	19.8	0.0	16.1	2.7	3.3	12.5	4.8	24.0
28a-Fhr	0.0	0.0	19.9	15.9	0.0	13.7	4.0	0.0	12.5	4.9	25.9
28b-1hr	0.0	0.0	15.0	15.4	0.0	11.8	2.2	0.0	8.7	3.4	16.6
28c-2hr	0.0	0.0	18.0	19.4	0.0	11.8	2.7	1.5	9.1	0.0	23.4
28d-3hr	0.0	0.0	19.6	15.3	0.0	10.7	2.0	2.0	10.1	0.0	19.5

28e-4hr	0.0	0.0	19.0	17.8	0.0	11.7	2.4	2.6	10.3	3.7	20.3
29a-Fhr	0.0	0.0	13.9	15.9	0.0	13.3	3.6	2.1	13.7	4.9	17.0
29b-1hr	0.0	0.0	17.3	18.4	0.0	11.2	2.5	3.0	8.3	4.0	17.0
29c-2hr	0.0	0.0	24.5	21.3	0.0	13.9	4.7	2.6	10.6	5.3	0.0
29d-3hr	0.0	0.0	20.0	19.3	0.0	12.9	3.5	1.8	9.4	4.7	17.1
29e-4hr	0.0	0.0	18.6	15.5	0.0	10.9	3.0	1.5	9.8	4.3	17.2

(AQuA) Auomated Quantification Algorithm
*In total 28 healthy Labrador Retriever dogs were sampled in a feed-challenge. The dogs are numbered 1-29 (nr 4 is missing). Fasting plasma samples were taken 15 minutes before serving of a test-meal (a-Fhr). Postprandial plasma samples were taken at 1 hour (b-1hr), 2 hours (c-2hr), 3 hours (d-3hr) and at 4 hours (e-4hr) after feeding.

Additional file 2B

Plasma concentrations (µM) of 11 of the in total 55 selected metabolites quantified with AQuA

Sample ID*	Acetate	Acetoacetate	Acetone	Alanine	Arginine	Asparagine	Betaine	Carnitine	Choline	Citrate	Creatine
01a-Fhr	27.3	12.9	3.7	155.7	86.5	25.6	140.0	9.2	3.2	154.2	6.0
01b-1hr	28.0	10.1	3.6	168.0	93.0	27.2	135.5	9.7	4.2	152.5	8.0
01c-2hr	29.1	10.7	3.8	178.1	89.3	29.5	144.4	9.8	4.1	139.1	8.1
01d-3hr	29.6	11.4	4.3	187.6	104.1	27.9	157.8	10.5	4.1	135.7	8.6
01e-4hr	28.8	13.5	3.9	180.7	95.2	29.0	152.1	9.6	3.5	129.2	7.9
02a-Fhr	24.6	5.9	2.9	160.0	85.3	26.8	141.3	4.5	2.8	98.5	5.6
02b-1hr	27.0	6.2	2.9	184.0	100.8	27.5	132.9	5.3	3.1	100.2	6.8
02c-2hr	34.6	9.5	3.2	302.9	120.8	35.4	146.1	4.8	4.0	116.2	7.0
02d-3hr	30.1	12.7	3.6	319.0	117.6	39.9	147.4	4.4	3.8	108.7	8.5
02e-4hr	29.1	7.9	3.1	264.7	101.3	34.2	153.9	4.8	3.4	105.2	8.1
03a-Fhr	27.9	9.0	3.6	179.9	93.0	26.4	244.8	9.5	2.7	151.9	5.6
03b-1hr	25.7	6.8	4.0	181.9	99.8	28.8	219.5	8.9	3.0	152.6	6.7
03c-2hr	26.0	7.5	4.4	259.0	105.5	33.5	216.0	9.0	3.7	139.1	7.4
03d-3hr	27.8	11.4	4.1	276.5	110.0	35.7	227.2	9.3	4.0	138.9	8.1
03e-4hr	30.4	12.4	3.9	267.4	101.5	34.8	226.1	9.0	3.4	134.2	7.7
05a-Fhr	22.2	3.1	3.1	174.2	94.3	21.2	97.7	10.0	3.0	82.5	9.3
05b-1hr	25.1	5.4	3.2	211.6	121.5	27.8	107.6	11.5	3.0	106.5	15.1
05c-2hr	28.5	7.9	3.3	208.9	129.0	29.9	102.6	11.5	3.2	112.4	17.3
05d-3hr	25.4	11.0	4.0	244.6	150.8	39.6	117.6	12.7	3.6	130.1	21.4
05e-4hr	25.1	10.2	4.0	191.6	130.4	33.0	116.8	12.7	3.1	128.2	21.2
06a-Fhr	27.6	7.2	4.2	238.3	94.4	26.6	82.1	8.1	2.2	143.9	3.4
06b-1hr	31.7	9.5	4.9	298.4	110.4	31.0	87.0	8.8	3.0	149.4	4.8
06c-2hr	29.3	10.4	3.6	258.3	98.4	27.2	85.3	8.5	3.1	126.4	6.3
06d-3hr	30.7	12.8	4.3	317.5	116.9	32.8	104.0	10.1	3.4	141.7	8.1
06e-4hr	27.6	10.6	3.9	283.3	104.5	29.9	100.1	8.7	3.1	129.1	5.9
07a-Fhr	26.3	9.9	3.3	194.1	90.2	33.7	101.1	4.7	2.7	113.5	5.6
07b-1hr	27.0	8.3	3.3	209.5	100.4	34.2	93.4	4.4	2.4	115.4	7.9
07c-2hr	28.7	9.7	3.5	262.0	115.6	38.5	102.6	5.3	3.6	125.3	9.3
07d-3hr	30.5	12.0	3.8	251.9	108.3	36.2	106.2	5.1	3.7	119.4	10.1
07e-4hr	27.7	9.3	3.6	235.9	103.1	37.4	104.2	5.2	3.6	115.5	8.6
08a-Fhr	27.8	5.7	3.0	183.8	81.2	24.4	64.7	13.6	2.8	119.6	7.7

08b-1hr	31.7	7.9	3.8	275.1	96.7	26.4	72.0	15.5	3.0	138.4	9.2
08c-2hr	31.2	9.0	3.8	306.5	102.8	33.2	80.2	16.2	3.3	140.5	9.9
08d-3hr	30.0	9.6	3.8	293.6	102.2	29.2	83.7	16.2	3.2	139.3	9.5
08e-4hr	29.6	9.6	3.9	253.0	88.6	26.8	85.6	15.9	2.7	132.9	9.7
09a-Fhr	26.2	4.9	4.0	236.5	96.2	27.6	38.5	16.4	3.3	123.2	6.8
09b-1hr	28.3	5.4	4.2	271.0	102.6	30.8	46.3	17.5	4.0	127.4	8.1
09c-2hr	35.0	6.1	4.0	312.3	108.2	31.1	58.0	19.2	4.4	132.4	8.4
09d-3hr	28.4	7.6	4.2	277.3	93.9	28.7	59.3	18.7	3.6	118.2	9.0
09e-4hr	27.8	6.6	4.1	233.0	88.0	28.4	62.3	19.3	3.3	117.9	8.6
10a-Fhr	25.3	0.0	3.1	240.8	79.5	21.0	27.1	7.4	2.9	100.7	3.5
10b-1hr	28.8	3.3	4.1	310.6	87.5	27.1	31.3	9.1	3.3	123.2	5.2
10c-2hr	26.4	3.7	5.2	318.8	97.7	28.6	33.7	8.3	4.2	118.0	5.9
10d-3hr	28.7	6.7	3.9	365.6	114.8	35.3	41.3	9.4	3.9	133.2	6.9
10e-4hr	28.8	7.0	3.2	315.3	96.9	28.8	36.3	8.3	3.2	110.1	5.8
11a-Fhr	30.6	11.1	3.6	184.8	112.4	27.7	112.3	13.1	3.2	149.0	21.8
11b-1hr	29.8	8.2	3.1	211.2	107.6	27.7	102.9	13.0	2.5	155.3	24.5
11c-2hr	29.1	12.4	4.4	238.8	110.1	26.4	102.1	12.7	2.7	150.5	30.2
11d-3hr	29.5	11.2	3.9	218.7	93.9	22.6	102.6	12.3	2.6	144.2	31.5
11e-4hr	28.9	14.0	4.1	207.4	99.2	23.0	103.9	12.1	2.5	115.5	30.3
12a-Fhr	29.7	6.2	3.4	234.1	108.9	26.5	73.4	8.2	4.4	111.3	6.7
12b-1hr	27.6	5.9	4.0	231.8	101.0	25.3	70.1	7.8	3.3	120.4	5.9
12c-2hr	26.5	6.3	3.5	219.8	101.7	22.9	64.9	7.1	3.9	111.7	6.1
12d-3hr	28.9	9.7	3.8	248.5	111.3	27.9	81.4	7.9	3.9	121.7	7.4
12e-4hr	27.9	8.7	4.8	231.8	104.1	29.6	83.5	7.8	3.3	121.1	7.2
13a-Fhr	30.1	4.1	3.1	221.8	112.2	26.3	76.3	26.8	3.0	114.4	53.5
13b-1hr	29.8	7.4	5.3	287.8	141.7	35.5	77.3	26.4	3.3	113.0	69.7
13c-2hr	31.1	9.8	4.5	311.4	156.5	37.7	88.1	26.3	3.9	117.8	70.3
13d-3hr	29.4	9.3	4.6	278.8	144.9	35.9	91.9	25.5	3.8	113.3	66.2
13e-4hr	27.5	8.8	3.7	262.8	140.5	32.8	98.6	26.0	3.1	104.9	61.8
14a-Fhr	25.6	5.8	3.7	180.2	100.9	26.7	110.2	8.0	4.2	115.5	4.8
14b-1hr	22.7	7.4	3.8	165.4	99.5	26.6	104.2	7.7	4.0	127.7	5.7
14c-2hr	24.0	5.4	4.5	182.6	94.1	27.7	101.3	7.7	4.0	125.2	5.2
14d-3hr	25.0	6.7	5.7	189.6	95.8	28.6	104.9	7.5	3.8	127.2	5.2
14e-4hr	22.2	7.2	6.9	169.8	98.4	25.3	105.6	7.3	3.5	120.2	5.0
15a-Fhr	28.8	10.8	4.4	334.1	100.7	28.1	161.2	17.3	3.2	157.8	6.3
15b-1hr	23.7	8.2	4.7	297.0	100.0	29.6	162.3	17.4	3.0	173.8	5.5
15c-2hr	23.4	7.3	4.6	300.4	95.7	25.2	157.6	16.8	3.0	160.2	5.4
15d-3hr	21.6	9.5	4.9	284.1	90.3	26.8	164.4	16.0	3.0	153.3	4.6
15e-4hr	23.3	11.0	5.0	330.1	98.6	27.6	168.2	18.0	3.5	148.4	6.5
16a-Fhr	24.4	2.9	3.5	232.0	86.2	26.3	41.6	14.0	3.6	120.1	3.4
16b-1hr	22.1	6.3	6.1	279.3	111.3	35.7	47.4	15.1	4.7	132.5	4.0
16c-2hr	28.1	8.1	5.3	321.9	123.9	39.1	62.8	17.6	5.0	153.9	4.4
16d-3hr	23.7	8.0	4.7	286.6	104.2	35.5	57.4	16.0	3.6	130.3	3.5
16e-4hr	23.2	6.0	4.8	269.2	98.6	32.5	57.4	15.5	3.3	122.7	3.6
17a-Fhr	23.8	0.0	3.5	151.8	85.2	26.4	204.8	7.2	7.9	120.3	8.2
17b-1hr	21.8	5.7	4.2	191.8	100.9	31.6	189.3	7.3	5.1	118.6	9.0
17c-2hr	22.7	6.9	5.0	195.5	103.5	30.3	187.5	7.4	4.7	117.3	9.9
17d-3hr	25.6	9.8	4.5	188.2	99.3	29.7	189.4	7.6	4.7	124.4	11.1

17e-4hr	20.6	13.0	5.7	161.9	102.8	28.6	176.9	7.3	3.8	116.9	10.4
18a-Fhr	22.6	13.6	6.7	232.7	98.4	30.5	191.8	7.1	3.2	153.7	14.0
18b-1hr	23.1	7.9	8.0	246.5	100.3	32.2	190.6	7.1	4.1	154.8	19.7
18c-2hr	21.2	12.8	11.2	252.2	103.9	32.7	200.1	7.6	4.3	148.9	22.4
18d-3hr	22.3	14.6	6.5	241.0	95.6	26.6	198.6	7.0	3.8	139.8	18.4
18e-4hr	23.2	15.5	8.0	211.9	87.3	26.9	196.4	7.7	3.5	152.0	18.6
19a-Fhr	20.2	3.6	3.9	197.7	90.6	25.8	67.8	18.3	4.3	114.0	3.9
19b-1hr	20.7	4.0	4.4	221.4	107.3	30.6	66.7	18.9	4.2	129.0	4.2
19c-2hr	22.5	6.9	4.4	225.4	101.1	30.3	70.5	19.6	4.7	139.8	5.7
19d-3hr	20.7	6.6	5.2	267.4	118.7	34.7	77.3	20.3	4.4	148.6	4.8
19e-4hr	22.0	7.8	4.7	216.2	111.8	32.8	75.3	19.8	4.3	143.4	5.7
20a-Fhr	21.3	4.7	4.5	157.0	79.8	20.5	66.6	5.0	3.8	102.3	8.6
20b-1hr	20.8	9.5	8.6	226.0	103.5	27.3	69.7	5.5	4.4	108.8	11.7
20c-2hr	22.7	9.4	8.2	211.3	97.4	27.1	74.8	5.4	3.7	96.4	12.2
20d-3hr	24.0	9.1	7.2	201.0	99.7	27.8	80.4	5.3	3.5	101.0	12.6
20e-4hr	22.1	8.2	5.4	171.9	93.2	24.1	82.0	6.1	2.8	94.0	10.6
21a-Fhr	22.7	2.5	3.3	255.9	103.5	34.5	74.6	20.2	3.0	119.4	5.2
21b-1hr	20.8	5.3	6.1	258.7	106.5	34.5	71.8	21.0	3.0	131.1	8.2
21c-2hr	24.6	6.8	5.1	245.1	102.1	35.2	76.6	21.2	3.7	132.1	10.3
21d-3hr	20.7	5.5	5.2	265.8	99.4	28.4	82.0	20.5	3.1	124.3	8.7
21e-4hr	22.7	8.7	6.1	230.1	101.2	29.5	82.7	21.3	3.0	131.9	9.0
22a-Fhr	20.6	7.9	4.5	247.9	88.0	27.8	74.5	18.6	3.5	156.2	4.6
22b-1hr	22.1	9.6	4.1	260.7	88.2	29.6	71.8	18.8	3.9	169.7	5.5
22c-2hr	20.7	12.0	5.3	302.5	95.6	31.4	79.7	19.5	4.8	189.3	5.8
22d-3hr	23.6	15.3	4.3	288.0	89.3	32.7	78.5	17.6	4.2	175.9	6.0
22e-4hr	22.8	25.5	4.7	278.8	102.5	29.2	80.6	18.6	3.8	179.1	6.7
23a-Fhr	21.6	5.2	5.3	219.3	81.7	23.5	45.3	14.6	3.4	151.6	5.1
23b-1hr	21.6	8.0	5.7	258.2	97.6	29.4	51.4	15.4	4.0	152.3	6.3
23c-2hr	24.5	9.6	4.5	245.1	88.1	22.9	53.8	14.9	3.9	142.7	7.2
23d-3hr	22.9	10.8	4.5	235.3	87.1	24.5	52.9	14.3	3.1	148.0	5.3
23e-4hr	23.7	11.7	5.6	212.4	79.8	22.5	51.7	14.1	3.0	147.5	6.4
24a-Fhr	18.7	4.2	5.8	195.0	101.3	26.3	187.4	12.9	6.7	126.6	10.9
24b-1hr	21.7	6.8	5.9	214.9	105.9	31.4	178.2	12.7	3.3	126.0	11.7
24c-2hr	20.1	6.7	6.6	238.2	104.5	29.5	184.0	13.0	3.4	120.9	12.6
24d-3hr	23.1	7.1	5.0	244.6	106.0	30.6	184.1	13.0	3.2	117.6	13.6
24e-4hr	23.2	7.3	5.3	247.9	103.8	31.3	195.9	13.9	3.7	121.8	14.1
25a-Fhr	20.5	4.2	3.7	266.8	102.8	27.3	108.8	13.8	2.4	86.1	14.2
25b-1hr	23.1	7.7	5.4	365.5	132.6	36.2	121.7	14.9	3.1	101.7	21.0
25c-2hr	20.8	8.8	7.5	342.9	126.6	36.3	134.5	15.1	3.3	107.1	23.1
25d-3hr	20.6	10.5	6.6	301.9	126.8	39.4	139.9	14.9	3.5	106.3	24.6
25e-4hr	24.0	10.5	5.2	273.2	113.2	32.1	142.8	14.6	2.8	99.4	22.7
26a-Fhr	25.3	12.7	4.1	239.1	106.1	21.7	54.2	9.9	3.2	156.2	10.0
26b-1hr	26.1	14.2	4.8	276.0	132.8	28.2	61.8	9.7	4.1	151.9	12.4
26c-2hr	22.8	11.4	5.8	272.8	123.3	26.0	69.7	10.0	4.1	150.9	13.1
26d-3hr	21.4	13.1	6.2	243.6	118.5	26.5	67.6	9.5	4.0	143.9	13.5
26e-4hr	22.4	12.9	6.3	218.8	117.0	29.1	69.6	10.0	4.3	134.7	11.4
27a-Fhr	24.5	4.6	3.7	349.1	104.9	25.7	183.4	7.4	3.6	135.2	11.4
27b-1hr	23.8	6.0	4.1	325.4	101.5	28.7	153.9	6.8	2.9	141.5	15.5

27c-2hr	23.2	7.3	4.6	306.8	89.9	23.8	154.8	7.2	3.6	144.2	18.4
27d-3hr	24.7	9.9	4.4	283.6	90.4	24.8	152.1	6.8	3.7	141.3	17.1
27e-4hr	22.4	8.7	5.9	271.2	86.9	25.0	145.4	7.2	3.3	146.9	17.4
28a-Fhr	26.2	8.2	3.8	308.1	98.4	29.8	161.5	7.4	3.4	148.5	11.4
28b-1hr	22.2	5.5	4.0	232.5	82.1	23.0	136.3	6.1	2.7	144.7	11.5
28c-2hr	25.5	6.1	4.5	235.2	78.9	22.8	133.1	6.2	3.1	150.6	13.1
28d-3hr	25.6	8.0	4.9	258.5	86.2	25.7	132.7	6.2	3.1	140.5	12.8
28e-4hr	24.1	9.3	4.6	239.5	85.8	24.3	122.2	6.2	3.1	132.6	12.0
29a-Fhr	19.6	4.8	3.6	227.7	72.3	25.6	50.2	10.2	2.7	130.2	2.9
29b-1hr	21.9	6.2	4.1	309.7	82.0	30.6	55.0	10.8	4.3	129.2	3.2
29c-2hr	22.7	9.9	5.4	351.8	99.7	33.6	73.2	12.9	4.6	130.5	3.8
29d-3hr	22.4	8.8	4.9	342.1	91.9	34.5	69.1	11.7	3.9	124.0	3.9
29e-4hr	20.9	11.1	5.2	317.1	88.4	35.7	71.6	11.8	3.2	119.3	4.2

(AQuA) Auomated Quantification Algorithm
*In total 28 healthy Labrador Retriever dogs were sampled in a feed-challenge. The dogs are numbered 1-29 (nr 4 is missing). Fasting plasma samples were taken 15 minutes before serving of a test-meal (a-Fhr). Postprandial plasma samples were taken at 1 hour (b-1hr), 2 hours (c-2hr), 3 hours (d-3hr) and at 4 hours (e-4hr) after feeding.

Additional file 2C
Plasma concentrations (μM) of 11 of the in total 55 selected metabolites quantified with AQuA

Sample ID*	Creatinine	Dimethylgly-cine	Dimethylsul-fone	Ethanol	Formate	Glucose	Glutamate	Glutamine	Glycerol	Glycine	Histidine
01a-Fhr	58.2	2.0	5.6	13.2	16.7	2824.8	0.0	407.8	263.4	133.9	45.0
01b-1hr	57.6	2.1	5.6	192.8	16.7	3018.2	0.0	375.1	407.1	161.8	46.0
01c-2hr	56.3	2.5	5.4	66.9	18.0	2902.4	0.0	330.5	274.4	166.3	45.3
01d-3hr	56.9	2.9	6.1	32.9	17.7	2934.9	0.0	337.7	219.9	175.8	49.3
01e-4hr	55.5	2.9	5.6	11.8	17.5	3147.8	0.0	331.7	319.0	169.7	48.3
02a-Fhr	41.2	3.4	6.1	10.3	16.1	2432.7	0.0	490.1	89.7	190.4	48.2
02b-1hr	40.1	3.3	6.2	28.8	15.1	2355.9	0.0	473.1	68.0	202.1	46.6
02c-2hr	45.9	3.9	8.1	23.6	16.7	2842.1	0.0	494.4	613.5	250.0	51.8
02d-3hr	42.6	4.0	6.5	9.4	15.9	2600.0	0.0	458.0	219.8	267.4	57.8
02e-4hr	43.2	3.8	6.3	8.0	16.2	2797.1	0.0	467.4	145.2	234.7	55.8
03a-Fhr	55.7	4.6	6.3	6.9	16.7	2414.9	0.0	496.2	142.9	150.5	53.0
03b-1hr	53.2	4.3	6.0	6.6	14.9	2220.2	0.0	467.2	201.0	174.5	51.2
03c-2hr	51.9	4.2	6.1	4.7	15.8	2153.5	0.0	435.5	330.2	198.2	57.6
03d-3hr	50.2	4.5	6.4	5.5	16.4	2359.2	0.0	424.5	205.8	201.7	60.7
03e-4hr	50.6	4.8	6.2	5.7	15.7	2569.0	0.0	417.8	560.4	194.6	59.5
05a-Fhr	49.1	2.1	3.9	5.6	15.4	2358.0	0.0	343.8	135.1	203.2	40.8
05b-1hr	55.8	2.4	4.5	7.4	15.2	2545.3	0.0	381.3	235.6	231.3	46.7
05c-2hr	52.9	2.7	4.3	5.5	15.2	2282.7	0.0	357.0	220.0	231.5	46.3
05d-3hr	58.9	3.3	4.7	6.1	16.4	2708.6	0.0	388.2	75.2	266.4	56.1
05e-4hr	57.8	3.3	4.5	6.2	15.8	2466.8	0.0	378.2	185.5	216.2	53.9
06a-Fhr	60.4	1.9	12.5	6.3	16.1	2469.1	0.0	450.7	143.5	142.2	53.3
06b-1hr	62.1	2.1	13.2	16.7	16.6	2644.4	0.0	440.6	279.0	182.4	54.6
06c-2hr	51.5	1.9	11.0	7.8	15.3	2337.5	0.0	362.0	464.1	154.6	48.8

06d-3hr	58.4	2.5	12.9	8.4	15.6	2760.9	0.0	409.5	176.0	191.0	56.5
06e-4hr	57.8	2.8	11.7	7.0	15.9	2479.9	0.0	387.9	366.1	170.6	50.8
07a-Fhr	42.3	3.9	6.0	7.5	16.7	2553.8	19.1	564.1	303.1	130.2	63.4
07b-1hr	40.4	3.8	5.9	6.7	16.8	2348.8	17.2	516.3	253.3	161.8	61.2
07c-2hr	43.9	4.6	6.6	6.1	17.0	2683.9	16.2	501.0	402.3	197.2	70.3
07d-3hr	42.2	5.2	6.5	5.7	17.1	2704.3	19.5	453.4	300.3	186.4	65.9
07e-4hr	43.2	5.4	6.5	5.4	17.4	2708.5	17.0	461.0	312.9	178.6	64.9
08a-Fhr	44.2	2.4	659.6	7.8	14.7	2214.5	0.0	381.0	106.8	118.0	42.0
08b-1hr	50.6	2.7	731.2	16.8	16.7	2444.1	0.0	419.4	165.8	175.3	45.0
08c-2hr	50.7	3.0	721.1	10.4	16.6	2381.3	0.0	409.5	258.2	197.5	55.2
08d-3hr	49.8	3.1	709.2	8.8	15.7	2600.8	0.0	403.8	442.6	176.9	53.0
08e-4hr	48.0	3.2	692.2	5.4	16.0	2417.0	0.0	396.2	308.2	158.8	53.1
09a-Fhr	50.5	1.9	3.8	7.4	14.7	2457.7	0.0	414.0	154.0	138.0	43.2
09b-1hr	51.7	2.1	4.0	27.1	15.3	2243.0	0.0	403.4	74.1	159.0	48.4
09c-2hr	51.5	2.6	5.9	20.6	14.7	2339.3	0.0	415.9	142.1	168.9	51.0
09d-3hr	47.6	2.7	4.4	13.7	15.2	2245.5	0.0	383.1	146.1	159.6	47.2
09e-4hr	48.6	2.9	4.1	6.8	15.3	2285.9	0.0	385.9	471.4	129.6	47.7
10a-Fhr	37.5	1.4	3.6	7.2	14.2	2267.3	0.0	357.1	335.9	144.8	40.6
10b-1hr	45.6	1.7	4.2	18.2	14.5	2519.7	0.0	396.3	319.7	171.9	45.9
10c-2hr	41.5	2.1	3.6	8.4	16.0	2202.3	0.0	360.7	413.7	191.0	47.4
10d-3hr	45.5	3.1	4.2	7.2	16.4	2675.8	0.0	386.7	103.6	225.3	55.0
10e-4hr	38.7	2.8	3.6	5.3	15.0	2411.5	0.0	337.4	396.4	185.9	50.2
11a-Fhr	53.4	3.0	4.2	10.7	16.6	2633.8	0.0	479.9	376.9	119.4	54.8
11b-1hr	53.0	2.9	5.8	12.7	15.9	2476.2	0.0	406.3	320.7	124.3	51.4
11c-2hr	52.5	3.0	4.2	7.7	15.3	2434.6	0.0	374.9	231.4	131.1	52.9
11d-3hr	51.0	3.0	4.2	7.5	15.7	2386.5	0.0	357.2	324.6	116.4	50.5
11e-4hr	49.9	3.1	4.9	6.3	14.6	2502.9	0.0	360.7	156.5	110.2	41.6
12a-Fhr	53.3	2.2	3.5	5.4	15.5	2419.9	0.0	416.6	349.6	185.8	48.4
12b-1hr	54.0	2.2	3.7	5.8	15.0	2508.9	0.0	405.8	81.5	174.4	47.0
12c-2hr	46.8	2.0	2.8	5.3	16.4	2190.8	0.0	345.1	385.9	170.7	45.0
12d-3hr	49.6	2.6	3.8	5.3	15.3	2205.5	0.0	374.3	533.9	190.3	46.8
12e-4hr	48.1	2.7	3.0	4.7	16.4	2280.7	0.0	366.5	261.9	188.9	48.1
13a-Fhr	44.7	2.2	3.7	5.8	15.8	2475.6	0.0	412.3	167.9	152.7	55.2
13b-1hr	44.1	2.0	3.9	13.1	16.0	2510.9	0.0	437.7	459.7	193.9	64.7
13c-2hr	44.9	2.6	6.3	10.5	14.9	2613.3	0.0	426.1	172.0	220.7	66.8
13d-3hr	41.8	3.0	4.1	6.9	15.5	2634.8	0.0	406.8	340.6	190.0	65.0
13e-4hr	41.0	3.2	6.7	6.3	16.0	2889.5	0.0	415.8	202.3	186.3	62.6
14a-Fhr	55.3	4.4	3.7	9.7	15.8	2514.8	0.0	512.6	758.3	153.9	55.5
14b-1hr	55.6	4.5	3.5	25.5	15.8	2451.3	0.0	437.7	438.0	149.0	54.2
14c-2hr	53.0	4.4	3.5	7.2	14.7	2310.1	0.0	421.7	437.8	151.4	54.1
14d-3hr	54.2	4.8	3.6	7.0	15.4	2420.8	0.0	430.1	549.1	147.5	58.5
14e-4hr	54.0	4.9	3.7	5.1	16.2	2587.4	0.0	433.9	160.2	148.6	50.8
15a-Fhr	47.7	3.3	22.4	11.4	16.1	2713.1	0.0	354.0	898.9	138.9	51.2
15b-1hr	51.1	3.1	22.4	33.2	15.1	2467.8	0.0	410.0	364.0	136.9	50.0
15c-2hr	49.3	3.0	21.9	18.1	15.5	2434.2	0.0	364.0	675.9	137.7	45.7
15d-3hr	48.0	2.7	22.2	23.3	15.5	2208.9	0.0	409.9	559.0	128.7	44.4

15e-4hr	49.3	3.4	22.7	18.6	17.4	2770.3	0.0	363.7	927.6	143.7	53.7
16a-Fhr	54.8	2.0	3.2	6.7	16.0	2589.3	0.0	369.4	734.7	112.1	46.9
16b-1hr	55.4	2.4	3.3	13.4	16.5	2338.5	0.0	363.4	350.0	171.9	53.7
16c-2hr	60.3	3.5	3.9	7.3	16.2	2643.3	0.0	406.2	810.3	188.8	60.7
16d-3hr	53.6	3.5	3.6	5.8	16.3	2608.9	0.0	377.3	614.1	166.5	53.0
16e-4hr	51.1	3.2	3.4	5.1	14.7	2540.8	0.0	376.8	567.8	160.7	51.8
17a-Fhr	59.4	4.1	4.0	0.0	15.6	2489.6	0.0	464.1	798.3	177.2	49.3
17b-1hr	57.5	4.1	4.2	5.6	16.0	2393.9	0.0	428.6	98.2	208.1	52.4
17c-2hr	58.5	4.2	4.3	4.9	17.4	2569.3	0.0	396.2	604.2	195.7	53.5
17d-3hr	57.1	4.5	4.4	5.0	16.5	2294.2	0.0	374.4	879.9	183.3	54.0
17e-4hr	53.9	4.6	5.4	7.2	16.3	2823.2	0.0	356.5	260.2	173.8	48.2
18a-Fhr	49.4	4.3	2.4	9.0	16.3	3029.6	0.0	459.6	834.2	149.7	50.3
18b-1hr	48.2	4.3	2.2	32.3	16.2	2411.7	0.0	409.6	295.3	181.8	55.0
18c-2hr	48.0	4.5	2.4	36.0	16.1	2755.8	0.0	392.0	591.9	171.3	59.7
18d-3hr	45.6	4.6	2.4	7.5	17.0	2911.4	0.0	380.7	811.4	144.4	46.6
18e-4hr	45.8	4.9	2.4	8.1	16.6	3600.9	0.0	393.5	251.2	153.1	56.5
19a-Fhr	58.3	3.1	3.4	6.4	15.3	2229.5	0.0	476.0	637.8	137.1	45.5
19b-1hr	59.5	3.0	3.6	26.3	15.9	2223.7	0.0	464.6	754.4	175.3	48.7
19c-2hr	60.4	3.5	3.6	10.6	16.2	2341.2	0.0	424.3	903.1	174.5	50.8
19d-3hr	60.3	4.0	3.7	6.7	16.6	2297.5	0.0	440.1	314.9	209.6	54.8
19e-4hr	58.6	4.2	3.7	6.5	14.4	2572.9	0.0	430.0	926.8	173.2	54.7
20a-Fhr	46.5	1.9	2.6	0.0	16.2	2446.9	0.0	386.3	78.2	161.3	46.0
20b-1hr	48.9	2.1	2.8	0.0	15.5	2672.8	0.0	390.9	320.0	208.5	47.7
20c-2hr	44.7	2.4	2.8	0.0	15.4	2308.9	0.0	358.1	56.5	209.2	51.5
20d-3hr	45.3	2.8	2.8	0.0	15.6	2582.9	0.0	373.8	265.4	190.1	54.2
20e-4hr	44.0	2.6	3.3	0.0	15.6	2848.5	0.0	384.2	75.7	161.2	50.1
21a-Fhr	51.8	2.2	4.3	4.9	14.1	2481.7	0.0	460.2	198.1	162.0	50.6
21b-1hr	52.1	2.1	3.8	20.4	14.8	2666.3	0.0	444.7	66.1	172.8	48.1
21c-2hr	51.7	2.3	3.6	6.6	14.6	2514.5	0.0	398.8	294.7	168.8	52.0
21d-3hr	50.4	2.5	3.5	5.0	14.0	2575.3	0.0	382.2	141.2	161.0	42.0
21e-4hr	50.2	2.7	3.7	0.0	15.7	2998.3	0.0	391.5	301.5	151.7	40.5
22a-Fhr	55.6	2.6	4.5	4.8	17.5	2394.3	0.0	355.1	227.5	118.4	41.8
22b-1hr	57.7	2.8	4.7	3.8	16.9	2425.7	0.0	323.5	161.4	136.9	45.8
22c-2hr	61.9	3.3	4.8	5.9	16.9	2489.8	0.0	336.3	364.4	148.9	46.0
22d-3hr	56.0	3.4	4.6	0.0	16.5	2264.8	0.0	301.8	260.4	146.0	47.2
22e-4hr	56.5	3.7	5.1	0.0	15.3	2771.4	0.0	308.3	198.6	143.9	42.9
23a-Fhr	57.9	2.0	5.0	4.9	14.9	2608.7	0.0	372.0	239.4	115.7	47.5
23b-1hr	61.3	2.3	5.3	3.6	15.4	2592.7	0.0	374.3	93.2	150.3	53.4
23c-2hr	57.0	2.6	5.3	3.6	16.2	2401.2	0.0	337.9	324.8	129.3	46.3
23d-3hr	56.4	2.9	5.1	0.0	15.1	2542.8	0.0	341.1	51.6	126.3	49.4
23e-4hr	56.2	2.9	5.1	0.0	15.4	2746.4	0.0	342.7	309.9	109.5	46.9
24a-Fhr	60.7	2.0	2.6	11.0	18.2	2214.2	0.0	547.8	307.8	160.6	58.4
24b-1hr	59.0	2.2	2.5	17.7	17.0	2830.3	0.0	525.8	185.2	179.3	55.1
24c-2hr	58.5	2.2	2.6	22.2	16.8	2734.4	0.0	514.4	49.1	191.8	52.5
24d-3hr	56.9	2.3	2.4	0.0	16.3	2890.5	0.0	497.5	311.3	187.7	59.0
24e-4hr	59.5	2.5	2.9	4.8	15.2	3156.9	0.0	532.2	220.5	197.1	56.4

25a-Fhr	47.4	1.3	6.0	6.6	14.6	2441.9	0.0	359.2	261.8	152.9	47.4
25b-1hr	51.1	1.6	6.2	21.7	16.9	2626.2	0.0	384.6	292.9	221.7	44.3
25c-2hr	50.2	1.9	6.3	16.7	15.5	2879.0	0.0	356.5	205.9	226.2	58.5
25d-3hr	47.6	2.3	6.2	6.7	16.2	2821.1	0.0	332.9	177.5	222.8	55.1
25e-4hr	47.0	2.5	6.0	0.0	16.5	3027.0	0.0	329.5	301.4	199.7	50.0
26a-Fhr	60.4	2.2	3.9	9.5	14.6	3142.1	0.0	347.6	213.9	150.5	38.4
26b-1hr	58.0	2.5	3.9	4.6	15.7	2608.1	0.0	328.4	349.2	197.9	45.9
26c-2hr	57.9	2.8	4.3	0.0	15.1	2739.2	0.0	343.0	515.0	175.7	46.0
26d-3hr	53.6	2.8	3.9	0.0	14.7	2874.2	0.0	343.5	85.9	177.5	49.6
26e-4hr	54.0	3.0	4.3	3.6	16.6	2951.3	0.0	358.2	692.5	164.6	48.5
27a-Fhr	49.7	4.9	4.7	5.5	15.3	2840.2	0.0	567.4	331.2	152.6	58.5
27b-1hr	46.7	4.6	3.3	6.2	15.4	2594.2	0.0	449.9	138.8	154.2	51.6
27c-2hr	46.3	4.6	3.4	4.9	15.0	2639.0	0.0	431.3	454.8	134.8	45.9
27d-3hr	46.6	4.8	4.1	0.0	15.8	2718.9	0.0	415.4	195.4	123.8	52.7
27e-4hr	47.8	4.9	3.3	0.0	16.5	2836.8	0.0	421.2	126.2	119.4	53.0
28a-Fhr	52.5	4.7	5.6	8.7	15.5	2664.9	0.0	397.9	305.8	163.7	53.9
28b-1hr	49.4	4.2	4.9	51.0	16.4	2455.6	0.0	341.1	300.1	116.1	45.9
28c-2hr	50.1	4.0	4.7	17.1	17.1	2510.5	0.0	355.8	648.2	111.5	50.3
28d-3hr	52.0	3.8	4.8	7.6	15.0	2526.6	0.0	375.1	604.9	106.2	53.8
28e-4hr	48.4	3.7	4.8	6.3	15.6	2440.9	0.0	351.2	304.0	110.4	50.6
29a-Fhr	60.9	2.0	2.2	7.4	15.8	2555.2	0.0	422.2	202.6	158.4	47.9
29b-1hr	59.4	2.2	2.4	7.5	16.8	2425.3	0.0	394.6	322.7	183.0	55.0
29c-2hr	60.1	2.8	3.2	6.1	17.5	2915.5	0.0	425.5	208.4	192.9	57.9
29d-3hr	57.4	2.8	2.5	0.0	15.7	2869.7	0.0	411.2	193.3	199.7	60.5
29e-4hr	56.6	3.0	2.5	0.0	15.9	3033.8	0.0	408.4	509.2	205.2	57.2

(AQuA) Auomated Quantification Algorithm

*In total 28 healthy Labrador Retriever dogs were sampled in a feed-challenge. The dogs are numbered 1-29 (nr 4 is missing). Fasting plasma samples were taken 15 minutes before serving of a test-meal (a-Fhr). Postprandial plasma samples were taken at 1 hour (b-1hr), 2 hours (c-2hr), 3 hours (d-3hr) and at 4 hours (e-4hr) after feeding.

Additional file 2D

Plasma concentrations (µM) of 11 of the in total 55 selected metabolites quantified with AQuA

Sample ID*	Isocaproate	Isoleucine	Lactate	Leucine	Lysine	Methanol	Methionine	Myo_Inositol	O_phosphoch oline	Ornithine	Phenylalanine
01a-Fhr	1.7	31.8	490.1	58.6	79.0	171.3	38.6	15.7	1.2	5.8	35.4
01b-1hr	1.6	39.5	445.3	70.9	85.6	145.3	39.0	18.2	1.6	8.4	31.4
01c-2hr	1.8	41.6	439.4	74.5	77.4	146.9	37.3	19.1	1.6	9.2	31.1
01d-3hr	1.9	48.2	496.2	88.1	79.6	136.3	40.6	19.3	1.9	10.9	32.4
01e-4hr	1.6	54.5	451.7	95.3	70.5	154.6	40.2	17.8	1.5	9.7	31.3
02a-Fhr	1.8	32.7	472.6	62.2	78.0	141.8	36.5	10.0	1.1	9.1	33.7
02b-1hr	1.6	33.8	476.4	65.1	84.9	150.2	35.8	13.8	1.7	12.1	33.8
02c-2hr	0.0	53.7	824.6	97.3	112.0	174.9	45.5	16.1	1.6	15.6	38.6
02d-3hr	0.0	59.4	839.2	104.5	106.0	158.9	43.6	14.5	1.7	17.5	35.7
02e-4hr	1.8	50.9	572.8	93.3	84.5	164.4	40.4	13.8	1.6	14.5	32.3
03a-Fhr	1.7	40.4	638.4	72.7	83.1	165.5	44.4	14.3	1.1	9.6	35.9

03b-1hr	1.6	37.1	536.2	66.3	81.3	138.8	40.1	14.7	1.2	10.9	31.5
03c-2hr	1.6	50.4	781.2	88.7	88.2	114.5	45.3	15.8	1.3	14.6	34.8
03d-3hr	2.0	57.2	660.3	101.1	80.6	133.7	45.6	15.1	1.5	15.8	35.0
03e-4hr	2.0	60.3	582.6	105.7	73.0	170.1	46.3	16.0	1.2	14.7	33.1
05a-Fhr	1.9	25.0	633.7	52.5	48.5	92.6	32.0	13.7	0.0	7.4	31.8
05b-1hr	1.6	35.2	580.8	65.5	68.8	150.7	38.4	18.4	1.2	11.6	38.1
05c-2hr	1.7	40.4	511.9	73.5	74.5	129.7	39.3	16.3	1.6	14.0	35.0
05d-3hr	1.8	52.8	591.9	95.4	88.9	149.4	42.5	19.1	1.4	18.1	40.9
05e-4hr	1.8	49.5	469.8	90.2	71.1	167.5	39.1	17.8	1.5	15.3	36.9
06a-Fhr	2.0	48.0	839.2	86.8	105.7	119.4	39.5	18.0	1.0	8.0	36.6
06b-1hr	2.1	54.3	824.1	96.8	123.8	149.7	41.1	20.4	1.4	11.7	35.2
06c-2hr	0.0	47.6	680.8	86.0	99.8	139.7	36.0	20.5	1.7	12.8	30.1
06d-3hr	2.1	61.9	738.7	108.6	114.0	165.1	41.4	22.1	2.2	16.8	35.4
06e-4hr	1.9	61.4	617.4	101.1	98.4	141.0	38.8	16.8	1.3	13.4	31.8
07a-Fhr	2.0	44.9	602.7	77.8	98.6	165.9	54.2	13.4	1.2	15.2	43.5
07b-1hr	1.7	45.1	448.8	77.5	111.1	159.3	51.2	14.4	1.4	18.1	40.3
07c-2hr	1.8	52.8	608.8	90.0	130.1	144.5	55.6	17.5	1.8	28.0	44.6
07d-3hr	1.9	51.6	630.1	89.2	121.0	166.5	52.7	17.2	1.7	31.0	41.8
07e-4hr	1.9	54.2	538.0	91.9	112.5	114.2	52.4	14.8	1.7	30.3	41.9
08a-Fhr	1.7	33.3	533.6	57.4	56.5	157.5	32.2	15.3	0.9	8.1	27.2
08b-1hr	1.8	41.7	682.0	72.4	74.0	176.1	36.4	18.9	1.2	11.9	29.3
08c-2hr	1.8	49.0	619.6	82.4	74.4	156.7	39.5	22.2	1.7	16.7	29.6
08d-3hr	2.0	50.6	721.0	86.4	74.0	160.9	38.7	19.7	1.5	16.0	30.4
08e-4hr	1.8	47.1	563.4	83.9	57.4	129.5	37.2	17.3	1.3	12.5	27.5
09a-Fhr	1.6	32.8	645.7	58.9	72.1	126.0	41.9	12.4	1.1	7.5	31.5
09b-1hr	1.8	40.8	474.6	69.8	78.7	149.0	41.5	17.4	1.5	11.6	30.6
09c-2hr	1.8	45.5	566.0	80.4	83.5	136.1	42.8	16.9	1.3	13.4	30.5
09d-3hr	1.7	45.5	561.1	79.5	73.9	142.9	38.1	16.6	1.6	13.8	29.7
09e-4hr	1.7	44.4	480.5	79.0	61.4	163.8	38.1	13.5	1.5	11.9	28.1
10a-Fhr	1.6	28.0	495.0	54.0	53.1	115.4	35.5	16.1	0.9	0.0	25.2
10b-1hr	0.0	34.5	556.4	62.3	62.6	139.8	39.8	18.0	1.4	8.6	28.5
10c-2hr	1.7	39.9	799.9	72.8	68.0	39.8	41.3	18.8	1.5	11.5	29.0
10d-3hr	1.7	50.6	762.8	90.4	78.1	135.7	47.5	20.2	1.9	15.1	32.5
10e-4hr	1.9	49.6	581.3	87.4	69.0	130.3	43.5	16.7	1.6	13.4	28.6
11a-Fhr	1.9	48.4	786.2	84.4	103.8	161.3	46.5	14.6	1.5	10.3	39.2
11b-1hr	1.6	44.7	933.6	78.0	101.9	153.6	44.6	20.0	1.4	10.6	36.8
11c-2hr	1.9	52.8	1051.0	90.5	92.5	112.3	43.2	18.3	1.7	12.1	34.7
11d-3hr	1.8	53.1	736.0	91.8	77.7	167.8	39.7	16.8	1.5	12.0	32.9
11e-4hr	1.7	58.4	712.0	101.7	81.4	173.2	41.1	17.6	1.3	11.5	33.0
12a-Fhr	1.8	34.4	933.0	62.5	72.3	160.1	40.3	19.2	1.5	9.8	33.6
12b-1hr	0.0	37.3	620.3	65.1	70.7	143.2	39.0	18.4	1.3	9.5	31.9
12c-2hr	1.7	37.3	513.5	67.1	69.7	160.0	35.0	17.1	1.4	11.5	29.1
12d-3hr	1.7	46.3	868.1	82.9	78.4	153.7	38.4	19.7	1.5	14.5	29.9
12e-4hr	0.0	45.7	562.8	81.1	68.8	137.8	36.7	17.2	1.5	13.5	28.1
13a-Fhr	0.0	32.4	776.7	56.5	86.5	151.8	48.6	15.7	1.0	10.0	29.5
13b-1hr	1.6	47.9	629.7	80.7	114.4	140.3	52.6	18.1	1.5	15.9	33.1

13c-2hr	0.0	58.7	597.5	99.5	126.5	165.5	56.8	19.9	1.1	21.2	33.8
13d-3hr	1.7	58.3	626.9	97.6	100.0	169.9	52.3	16.2	1.7	19.7	30.2
13e-4hr	1.9	57.5	545.8	100.5	95.8	175.5	52.1	17.1	1.5	17.5	31.4
14a-Fhr	1.9	43.2	661.8	76.4	115.5	132.5	46.2	16.3	1.2	10.5	44.6
14b-1hr	0.0	45.9	546.7	82.3	106.6	185.3	42.1	16.7	1.1	14.9	41.4
14c-2hr	1.7	52.6	599.7	91.5	105.6	132.0	41.8	17.5	1.3	17.1	38.8
14d-3hr	1.9	58.5	711.4	102.1	101.0	131.9	40.3	16.0	1.5	17.0	38.8
14e-4hr	1.9	63.9	661.1	112.7	98.5	109.5	40.6	16.3	1.3	15.9	40.6
15a-Fhr	1.8	44.1	778.3	79.2	105.6	150.3	40.8	17.8	1.5	13.6	31.9
15b-1hr	1.8	37.4	606.7	70.8	107.6	115.9	40.9	17.8	1.6	9.9	32.5
15c-2hr	1.6	37.2	590.0	67.8	101.7	117.2	38.4	18.2	1.3	11.9	30.9
15d-3hr	1.6	33.7	928.2	65.7	103.8	549.0	38.0	18.5	1.1	8.3	31.7
15e-4hr	1.9	46.2	760.2	83.7	104.5	548.9	41.1	18.7	1.5	14.9	31.9
16a-Fhr	1.7	33.1	840.0	63.9	68.0	138.7	41.5	22.5	1.1	5.8	34.9
16b-1hr	1.8	48.1	684.5	88.1	82.2	100.0	44.4	25.2	1.7	11.7	35.6
16c-2hr	2.0	58.9	748.5	109.7	87.0	149.0	50.0	29.9	2.1	15.3	40.7
16d-3hr	1.8	57.6	420.3	103.4	70.5	134.7	45.2	21.6	1.6	12.8	35.5
16e-4hr	1.9	53.3	468.9	95.1	63.6	130.0	42.0	21.0	1.4	10.6	32.1
17a-Fhr	0.0	26.2	677.2	49.9	78.4	125.8	40.0	15.4	0.9	8.8	29.0
17b-1hr	1.5	34.8	551.2	61.9	85.0	140.6	40.6	18.4	1.2	11.6	28.2
17c-2hr	0.0	39.4	698.0	72.9	79.1	130.0	39.2	21.4	1.4	16.0	26.6
17d-3hr	1.9	43.6	697.6	82.0	75.3	149.2	37.3	23.1	1.6	19.7	26.9
17e-4hr	1.8	48.6	600.5	89.4	71.5	203.5	37.0	19.2	1.6	16.1	24.5
18a-Fhr	1.7	31.2	949.1	60.2	110.4	117.4	59.6	16.6	1.2	8.8	41.3
18b-1hr	1.7	37.2	716.8	69.2	107.0	126.2	56.3	18.0	1.6	14.0	35.6
18c-2hr	1.7	49.4	765.7	90.1	101.1	694.2	56.6	17.0	2.1	21.2	35.8
18d-3hr	1.9	55.6	753.6	100.2	88.9	147.7	53.1	16.2	1.8	21.2	32.2
18e-4hr	1.9	53.7	563.5	98.2	79.7	124.4	51.0	13.9	1.8	20.0	29.8
19a-Fhr	1.5	28.2	529.5	52.5	51.8	134.6	36.0	17.7	1.1	7.1	33.9
19b-1hr	1.7	37.1	470.4	67.6	66.1	129.1	38.3	19.6	1.4	10.8	33.7
19c-2hr	1.9	39.8	644.6	74.5	66.4	219.4	38.2	21.4	1.5	14.3	34.2
19d-3hr	1.9	47.9	540.4	89.1	72.5	132.6	38.4	22.2	1.4	15.3	33.4
19e-4hr	1.8	48.6	528.9	89.8	64.2	142.8	38.1	19.1	1.6	14.5	32.9
20a-Fhr	1.9	33.3	643.4	61.4	67.8	26.6	32.9	13.9	1.0	6.7	31.7
20b-1hr	1.9	46.0	640.1	85.5	87.1	61.0	37.6	19.9	1.5	12.1	32.7
20c-2hr	2.1	51.0	473.6	91.8	76.9	35.8	36.2	17.3	1.6	13.1	30.7
20d-3hr	2.2	55.4	408.8	100.5	73.3	35.1	37.1	14.6	1.4	12.8	31.5
20e-4hr	2.2	49.7	398.9	95.3	64.7	47.6	37.5	16.3	1.6	10.8	32.1
21a-Fhr	1.9	35.5	706.4	67.4	99.9	41.7	54.1	18.0	1.2	8.0	40.9
21b-1hr	2.0	39.4	609.2	71.3	97.0	27.9	50.6	17.4	1.6	11.7	36.1
21c-2hr	2.2	44.5	669.8	80.3	89.5	45.3	49.2	19.2	1.8	14.3	33.6
21d-3hr	2.2	50.2	603.0	89.5	81.2	51.5	46.6	17.7	1.3	13.3	33.5
21e-4hr	2.1	57.9	589.6	101.5	80.2	27.4	48.7	18.5	1.6	14.2	34.8
22a-Fhr	2.2	36.7	792.7	66.3	69.7	35.0	44.5	17.1	0.8	0.0	42.1
22b-1hr	2.1	42.0	734.0	75.0	71.1	39.2	44.0	21.9	1.3	11.0	39.5
22c-2hr	2.2	54.0	917.4	93.6	72.7	80.8	45.2	23.1	1.5	13.5	42.0

22d-3hr	2.3	61.0	882.8	103.0	66.9	40.4	42.4	21.0	1.5	14.9	37.9
22e-4hr	2.2	70.1	909.3	121.1	66.8	31.6	45.0	23.6	1.9	15.1	40.2
23a-Fhr	2.1	30.5	733.1	57.6	60.1	35.4	43.3	18.0	1.1	7.3	40.8
23b-1hr	2.3	42.7	612.8	76.7	70.6	38.1	44.0	22.8	1.6	13.1	39.4
23c-2hr	2.3	43.7	771.3	83.0	60.9	54.0	40.6	27.4	1.7	13.1	37.3
23d-3hr	2.1	46.6	642.0	86.7	55.6	33.8	38.0	20.0	1.7	12.1	34.0
23e-4hr	2.2	43.8	653.3	82.7	49.9	48.8	36.7	19.1	1.3	10.2	33.3
24a-Fhr	2.0	37.4	1868.0	65.6	89.7	61.1	38.2	20.7	1.0	8.3	41.8
24b-1hr	2.0	32.3	503.0	59.4	88.2	44.0	33.5	13.5	1.2	11.3	34.6
24c-2hr	1.9	36.3	519.7	65.4	87.1	32.9	33.5	15.7	1.2	12.8	34.0
24d-3hr	2.1	37.6	452.1	66.4	81.2	42.1	32.3	14.7	1.2	13.1	31.8
24e-4hr	1.8	42.3	542.4	75.4	84.0	39.7	36.3	13.9	1.5	13.9	33.0
25a-Fhr	1.9	30.1	837.9	56.2	92.8	26.2	35.7	13.0	1.1	8.5	30.1
25b-1hr	1.9	44.4	696.7	79.3	119.9	34.1	38.5	17.7	1.2	13.0	31.8
25c-2hr	1.9	46.0	631.7	83.9	109.6	30.0	38.0	19.2	1.3	14.7	28.6
25d-3hr	2.2	49.5	641.6	90.2	104.3	40.1	38.2	16.8	1.7	13.6	26.5
25e-4hr	2.0	47.1	581.4	85.6	89.2	55.7	37.1	15.6	1.3	12.2	24.5
26a-Fhr	2.2	35.5	1266.2	59.1	79.6	46.9	40.2	28.0	2.5	7.0	31.7
26b-1hr	2.0	45.8	927.8	78.2	99.8	42.8	41.7	29.0	2.9	14.6	31.5
26c-2hr	2.0	48.3	1000.6	84.2	86.8	41.1	41.7	27.2	2.8	14.4	32.0
26d-3hr	2.2	49.2	885.2	84.0	78.4	47.5	37.5	23.9	2.7	13.7	30.5
26e-4hr	2.2	50.5	740.5	88.2	74.0	42.0	40.9	22.2	2.9	13.5	30.0
27a-Fhr	2.0	36.1	1298.4	69.9	119.3	62.4	60.8	16.4	1.0	10.3	41.7
27b-1hr	2.1	40.0	926.4	70.9	106.1	66.0	54.1	17.5	1.2	14.1	34.2
27c-2hr	2.2	45.6	759.6	81.1	98.2	60.9	51.9	14.8	1.4	16.0	32.6
27d-3hr	2.2	49.1	1236.1	88.8	89.5	64.6	49.0	19.0	1.2	17.1	30.8
27e-4hr	2.1	46.4	888.4	82.4	77.9	44.5	46.9	15.4	1.1	15.9	32.6
28a-Fhr	1.8	39.1	820.6	76.6	86.2	43.2	52.7	22.9	1.6	13.2	37.6
28b-1hr	1.5	33.7	590.0	61.1	63.2	35.7	44.3	17.8	1.1	7.7	30.7
28c-2hr	1.8	34.2	651.7	63.8	61.9	39.8	40.1	19.6	1.1	8.1	29.8
28d-3hr	1.9	45.8	811.4	80.6	62.6	46.0	43.0	19.6	1.1	8.3	35.9
28e-4hr	1.7	44.2	850.6	77.9	56.8	43.7	39.9	18.8	1.3	8.3	32.5
29a-Fhr	1.7	35.3	710.2	60.9	64.4	54.0	37.8	15.9	1.0	4.9	32.5
29b-1hr	1.8	43.9	645.4	78.6	71.8	50.7	39.3	18.6	1.6	10.3	32.5
29c-2hr	2.6	54.9	696.0	102.1	81.3	58.4	44.6	21.7	2.7	15.8	39.4
29d-3hr	2.4	53.7	797.7	94.2	69.3	53.3	39.4	19.7	1.9	11.6	34.1
29e-4hr	2.4	56.4	569.2	97.1	64.6	51.7	40.6	18.0	1.7	10.4	33.5

(AQuA) Auomated Quantification Algorithm

*In total 28 healthy Labrador Retriever dogs were sampled in a feed-challenge. The dogs are numbered 1-29 (nr 4 is missing). Fasting plasma samples were taken 15 minutes before serving of a test-meal (a-Fhr). Postprandial plasma samples were taken at 1 hour (b-1hr), 2 hours (c-2hr), 3 hours (d-3hr) and at 4 hours (e-4hr) after feeding.

Additional file 2E

Plasma concentrations (µM) of 11 of the in total 55 selected metabolites quantified with AQuA

Sample ID*	Proline	Propionate	Pyroglytamate	Pyruvate	Sarcosine	Serine	Succinate	Threonine	Trimethylamine_N_oxide	Tyrosine	Valine
01a-Fhr	89.4	3.9	0.0	26.8	2.1	106.6	7.1	100.1	35.0	30.5	102.4
01b-1hr	129.5	3.9	0.0	36.6	2.3	109.9	5.7	103.4	35.5	28.7	115.3
01c-2hr	130.9	3.6	0.0	41.4	2.6	103.3	5.1	100.6	35.3	24.2	125.7
01d-3hr	151.1	0.0	0.0	38.7	3.3	108.2	5.7	107.2	37.3	25.6	144.2
01e-4hr	150.1	0.0	0.0	38.6	3.0	110.9	4.7	108.2	38.3	26.8	157.0
02a-Fhr	120.6	0.0	0.0	34.2	3.3	109.6	5.2	98.2	30.3	30.9	95.6
02b-1hr	138.2	4.2	0.0	31.3	3.3	107.3	5.4	103.0	25.0	29.8	95.9
02c-2hr	198.4	0.0	0.0	48.0	4.1	134.4	5.7	129.4	35.3	34.3	143.4
02d-3hr	218.6	0.0	0.0	65.3	4.3	139.0	4.1	143.1	32.0	33.3	162.0
02e-4hr	197.4	0.0	0.0	42.9	3.9	129.6	4.9	139.0	33.4	30.4	151.4
03a-Fhr	106.0	0.0	0.0	31.1	4.5	107.8	5.9	100.1	36.5	30.2	109.9
03b-1hr	113.3	0.0	0.0	33.7	4.4	101.3	4.9	93.6	33.4	24.8	104.5
03c-2hr	157.2	0.0	0.0	59.3	4.6	112.7	4.9	109.6	34.4	28.2	134.1
03d-3hr	178.9	0.0	0.0	61.0	5.2	118.4	4.1	119.1	35.4	29.4	157.7
03e-4hr	180.4	3.6	0.0	50.3	5.1	122.2	4.5	126.9	37.9	29.0	170.4
05a-Fhr	132.4	0.0	0.0	28.6	2.7	117.1	5.9	100.7	26.3	33.3	87.8
05b-1hr	162.3	0.0	0.0	38.9	2.7	127.3	6.4	128.1	33.7	40.1	105.7
05c-2hr	168.7	3.3	0.0	30.2	2.9	125.3	6.7	137.4	29.4	39.6	114.8
05d-3hr	211.2	0.0	0.0	40.9	3.9	148.2	6.2	164.1	32.0	49.7	150.9
05e-4hr	179.0	3.5	0.0	30.2	3.8	128.3	6.6	152.9	29.6	43.7	149.7
06a-Fhr	95.7	0.0	0.0	49.8	2.6	92.6	6.1	110.2	28.5	39.3	140.5
06b-1hr	139.8	0.0	0.0	50.8	2.5	101.3	5.6	126.1	27.9	40.3	156.7
06c-2hr	133.7	0.0	0.0	51.0	2.3	87.0	4.3	107.5	26.2	34.4	135.0
06d-3hr	170.9	6.1	0.0	65.0	3.2	102.6	5.1	128.6	29.0	40.8	172.5
06e-4hr	154.3	0.0	0.0	58.3	3.4	96.4	4.4	118.3	30.1	39.9	172.2
07a-Fhr	87.4	3.4	0.0	32.3	2.8	115.4	8.2	119.7	27.3	51.9	119.9
07b-1hr	115.9	0.0	0.0	39.2	2.6	120.1	5.9	123.3	24.1	49.8	122.9
07c-2hr	152.1	0.0	0.0	63.9	3.4	129.2	6.0	141.7	29.2	51.8	143.3
07d-3hr	145.0	3.7	0.0	49.7	4.1	125.9	5.1	134.7	29.0	51.7	146.0
07e-4hr	141.6	0.0	0.0	56.4	3.9	122.3	5.6	136.7	31.6	53.1	154.5
08a-Fhr	81.5	0.0	0.0	32.4	2.8	83.0	5.4	90.9	27.4	24.2	93.1
08b-1hr	136.2	0.0	25.6	55.3	3.0	104.0	4.5	109.5	26.2	26.9	114.8
08c-2hr	175.4	3.3	23.5	58.8	3.3	115.0	4.5	123.1	27.9	28.0	134.3
08d-3hr	170.8	0.0	0.0	62.7	3.8	105.3	4.7	115.2	30.8	28.0	143.7
08e-4hr	154.7	3.5	0.0	49.2	3.6	96.6	4.3	114.9	27.0	27.5	139.8
09a-Fhr	110.7	0.0	0.0	36.1	2.1	92.9	6.5	108.5	26.1	41.1	92.7
09b-1hr	142.2	3.4	0.0	29.9	2.2	92.3	6.5	119.5	25.0	41.6	106.6
09c-2hr	163.0	3.4	29.1	38.5	2.6	96.8	5.5	121.2	27.9	41.3	124.7
09d-3hr	162.8	0.0	0.0	55.9	2.8	98.6	4.6	113.5	25.1	38.8	129.5
09e-4hr	141.4	0.0	0.0	38.9	2.8	85.8	5.7	104.0	26.8	35.2	129.7

10a-Fhr	107.4	0.0	0.0	32.5	2.2	110.5	5.7	108.0	25.6	28.0	88.1
10b-1hr	129.3	0.0	0.0	29.6	2.0	111.5	6.5	120.1	24.1	30.5	107.3
10c-2hr	152.5	0.0	0.0	42.4	2.4	110.0	5.3	122.9	21.0	30.7	114.4
10d-3hr	191.0	0.0	0.0	60.8	3.7	126.2	5.5	139.1	24.9	36.0	143.5
10e-4hr	175.3	3.7	0.0	43.9	3.4	112.3	5.0	130.6	26.1	32.3	138.7
11a-Fhr	101.5	3.9	0.0	46.6	3.3	114.2	8.2	95.0	31.7	36.7	122.5
11b-1hr	120.2	0.0	0.0	43.6	2.6	100.6	7.0	96.6	31.8	33.6	119.5
11c-2hr	134.5	0.0	0.0	61.8	3.0	95.5	6.0	93.9	33.2	33.3	139.0
11d-3hr	121.4	0.0	0.0	51.8	3.2	89.6	5.4	84.4	30.4	31.2	147.9
11e-4hr	126.7	0.0	0.0	47.9	2.9	85.5	5.1	83.4	31.8	31.2	162.2
12a-Fhr	133.4	0.0	0.0	34.0	2.6	103.3	7.9	106.3	30.2	42.1	104.6
12b-1hr	132.7	0.0	0.0	31.9	2.4	92.0	7.6	108.8	30.1	43.0	106.6
12c-2hr	140.9	3.3	0.0	28.4	2.5	86.7	6.1	101.1	23.4	36.7	105.7
12d-3hr	169.1	0.0	0.0	39.1	3.2	98.8	6.2	107.6	28.1	39.4	133.2
12e-4hr	167.4	0.0	0.0	30.9	3.4	95.2	6.5	110.9	24.6	36.7	133.6
13a-Fhr	134.2	0.0	0.0	38.0	2.3	109.0	6.1	127.2	27.9	30.9	92.8
13b-1hr	188.9	0.0	0.0	36.3	2.2	122.8	5.0	150.3	28.1	37.5	122.3
13c-2hr	231.0	0.0	0.0	39.6	3.0	135.1	4.5	157.6	31.4	43.9	156.9
13d-3hr	216.4	0.0	0.0	46.6	3.6	122.7	4.7	150.1	30.2	40.3	162.7
13e-4hr	222.9	0.0	0.0	38.1	3.4	131.0	4.5	151.9	35.1	37.3	170.2
14a-Fhr	109.6	0.0	30.9	35.9	3.8	95.3	7.2	108.5	31.2	37.0	122.0
14b-1hr	115.5	0.0	0.0	37.2	4.3	84.9	5.1	106.8	30.3	33.5	128.4
14c-2hr	130.9	0.0	26.5	31.4	4.0	83.5	5.6	107.2	28.5	32.7	144.7
14d-3hr	141.0	0.0	0.0	44.7	4.1	84.1	5.9	109.4	28.2	31.5	161.3
14e-4hr	131.7	0.0	0.0	40.9	4.5	88.6	5.4	100.6	31.4	32.6	178.7
15a-Fhr	142.8	0.0	0.0	72.9	4.3	91.3	4.2	90.1	32.8	34.3	128.1
15b-1hr	125.2	0.0	0.0	50.8	3.9	93.6	4.3	87.9	33.3	33.7	109.2
15c-2hr	126.8	0.0	25.8	57.5	4.0	90.6	3.4	83.3	31.5	31.7	110.1
15d-3hr	114.5	0.0	0.0	65.3	3.7	97.3	4.6	85.7	31.5	34.7	101.0
15e-4hr	153.9	0.0	0.0	61.5	4.6	94.1	3.9	92.7	34.3	34.9	137.6
16a-Fhr	112.6	0.0	0.0	36.9	2.1	78.2	7.5	114.1	23.9	35.3	104.0
16b-1hr	166.0	0.0	0.0	47.9	2.6	94.2	5.6	129.6	26.0	36.2	133.4
16c-2hr	204.8	0.0	35.8	55.0	3.5	106.8	6.1	141.1	27.7	40.2	167.8
16d-3hr	182.3	0.0	0.0	33.1	3.4	98.5	5.9	126.0	28.0	35.8	168.0
16e-4hr	178.6	0.0	0.0	45.8	3.0	98.2	5.7	124.9	25.8	35.6	162.1
17a-Fhr	114.4	0.0	0.0	37.7	4.9	102.2	5.8	104.3	36.4	31.2	84.7
17b-1hr	150.4	0.0	0.0	44.2	4.5	107.3	4.8	120.9	34.5	31.5	99.6
17c-2hr	169.1	0.0	0.0	41.4	4.9	105.9	4.9	117.4	38.1	30.2	115.1
17d-3hr	174.3	0.0	0.0	38.3	5.4	103.9	4.8	119.5	34.4	30.4	135.0
17e-4hr	162.5	0.0	0.0	40.7	5.2	101.9	4.2	112.2	41.7	28.4	146.8
18a-Fhr	98.5	0.0	0.0	56.5	2.5	137.9	7.0	159.1	41.3	46.3	110.8
18b-1hr	132.7	0.0	0.0	68.4	2.8	130.3	5.6	158.3	33.1	43.4	122.4
18c-2hr	148.0	0.0	0.0	67.3	3.2	128.0	5.2	162.0	37.5	42.1	147.3
18d-3hr	140.8	0.0	0.0	38.6	3.0	117.0	5.0	144.8	38.1	41.9	163.6
18e-4hr	131.3	0.0	0.0	49.4	3.7	118.3	5.3	142.4	43.9	38.0	165.6
19a-Fhr	101.1	0.0	0.0	37.3	3.0	100.2	5.9	88.8	24.0	36.0	83.4

19b-1hr	140.2	0.0	0.0	31.1	3.0	105.9	5.2	100.1	25.1	37.4	101.7
19c-2hr	156.3	0.0	0.0	46.5	3.3	109.6	4.5	103.7	25.1	35.6	116.0
19d-3hr	192.7	0.0	25.8	38.2	3.7	121.1	5.1	118.2	25.2	38.5	140.9
19e-4hr	168.9	0.0	0.0	39.3	3.9	112.8	5.7	111.2	27.5	38.3	144.0
20a-Fhr	93.6	0.0	0.0	38.8	2.0	85.6	5.1	70.5	24.1	26.6	101.5
20b-1hr	158.6	0.0	0.0	48.5	2.5	107.4	3.9	91.0	27.0	30.6	133.2
20c-2hr	158.9	0.0	0.0	34.6	2.8	99.7	3.8	91.5	25.7	27.0	147.6
20d-3hr	157.3	0.0	0.0	29.0	3.1	101.2	4.5	94.3	26.8	27.3	164.5
20e-4hr	139.6	0.0	0.0	22.4	2.6	93.9	5.0	87.5	26.9	28.3	151.4
21a-Fhr	130.9	0.0	0.0	43.6	2.6	96.8	6.2	138.1	28.8	37.3	113.7
21b-1hr	147.9	2.6	0.0	51.2	2.5	96.6	5.4	143.5	29.4	34.0	116.0
21c-2hr	155.6	0.0	0.0	60.1	2.7	91.1	5.1	139.0	28.4	29.7	127.8
21d-3hr	162.2	0.0	0.0	47.4	2.6	90.5	4.5	129.0	27.7	31.2	147.0
21e-4hr	164.0	0.0	0.0	51.9	3.0	91.9	4.6	132.8	34.9	32.4	163.0
22a-Fhr	98.4	0.0	0.0	56.9	2.8	100.4	5.1	129.4	29.1	35.2	110.3
22b-1hr	124.5	0.0	0.0	62.7	2.6	99.5	5.2	130.9	27.6	34.5	120.1
22c-2hr	142.7	0.0	0.0	81.1	3.5	103.2	5.0	129.8	31.0	37.1	154.4
22d-3hr	149.2	0.0	0.0	80.0	3.6	96.5	4.7	123.0	26.3	31.7	168.1
22e-4hr	164.3	0.0	0.0	81.9	3.8	96.0	4.7	123.9	36.6	31.6	193.2
23a-Fhr	90.9	0.0	0.0	50.4	2.5	91.5	5.6	110.3	26.9	40.6	99.5
23b-1hr	135.0	3.2	0.0	59.3	2.9	99.6	5.0	122.8	24.3	41.6	126.6
23c-2hr	130.4	0.0	0.0	58.8	3.2	87.5	4.8	104.3	28.9	35.8	135.9
23d-3hr	128.4	0.0	0.0	51.1	3.2	84.5	5.3	101.9	28.2	35.4	143.7
23e-4hr	121.0	0.0	0.0	56.3	3.1	80.7	4.5	94.3	27.9	33.1	140.6
24a-Fhr	111.4	0.0	26.7	49.1	3.4	111.0	8.7	188.5	33.8	48.8	100.9
24b-1hr	112.9	0.0	0.0	35.9	3.9	107.6	5.3	188.0	34.3	43.1	96.4
24c-2hr	142.6	0.0	0.0	47.8	3.7	114.4	5.1	183.7	37.6	40.8	106.7
24d-3hr	141.7	0.0	0.0	43.2	4.1	111.8	5.1	189.2	35.7	39.9	112.6
24e-4hr	146.6	0.0	0.0	43.5	4.2	116.4	5.6	192.4	41.1	43.2	126.2
25a-Fhr	108.6	0.0	0.0	51.0	2.4	120.1	4.5	111.3	29.4	39.1	89.9
25b-1hr	181.8	0.0	0.0	51.4	2.6	147.4	3.8	132.7	31.3	44.1	122.6
25c-2hr	197.5	0.0	0.0	48.6	2.8	142.2	3.5	138.7	36.2	39.5	135.5
25d-3hr	195.6	0.0	0.0	48.9	3.0	143.8	4.1	138.4	38.3	37.4	151.2
25e-4hr	182.8	0.0	0.0	47.6	2.8	135.1	3.4	129.4	34.4	35.1	146.5
26a-Fhr	101.0	0.0	0.0	100.5	2.3	84.2	6.3	101.1	31.3	36.8	105.9
26b-1hr	162.3	0.0	0.0	85.3	2.9	95.7	5.1	120.1	29.5	36.7	134.4
26c-2hr	154.0	0.0	0.0	81.8	3.4	93.0	5.3	115.3	30.0	34.0	145.3
26d-3hr	154.0	0.0	0.0	85.1	3.5	97.1	4.6	107.8	32.3	28.8	148.6
26e-4hr	152.2	0.0	0.0	61.2	3.2	97.7	4.6	115.4	33.0	28.4	155.8
27a-Fhr	142.0	0.0	0.0	73.6	3.4	120.2	7.8	145.6	42.4	42.0	125.7
27b-1hr	147.1	0.0	0.0	69.4	3.2	100.5	4.9	137.4	30.8	39.4	123.9
27c-2hr	147.3	0.0	0.0	64.7	3.1	94.7	4.5	124.8	33.0	38.3	137.4
27d-3hr	139.7	0.0	0.0	93.7	3.6	91.0	4.7	115.7	36.0	37.3	147.7
27e-4hr	124.5	0.0	0.0	80.6	3.5	91.1	4.6	112.5	35.6	36.1	141.8
28a-Fhr	138.5	4.5	0.0	65.3	4.8	113.9	4.8	176.1	34.3	36.9	120.7
28b-1hr	100.9	0.0	0.0	51.6	3.5	84.8	4.3	138.2	32.1	30.1	108.5

28c-2hr	102.6	0.0	0.0	55.5	3.7	90.0	3.9	135.2	32.3	29.4	110.0
28d-3hr	111.9	0.0	0.0	43.9	2.8	87.3	4.9	144.3	30.8	32.7	132.8
28e-4hr	108.5	0.0	0.0	57.4	2.9	82.5	4.6	133.5	31.7	33.1	129.4
29a-Fhr	98.1	0.0	0.0	58.4	2.5	91.2	4.9	88.3	24.8	32.4	93.8
29b-1hr	139.5	0.0	0.0	61.1	2.7	104.8	4.3	94.5	26.5	34.8	120.0
29c-2hr	178.9	0.0	0.0	68.3	3.4	119.6	4.6	103.1	29.2	37.4	149.1
29d-3hr	176.0	0.0	0.0	73.6	3.2	119.3	4.8	107.5	31.4	32.6	153.2
29e-4hr	184.2	0.0	0.0	62.9	3.1	126.5	3.8	111.5	31.0	35.3	165.0

(AQuA) Auomated Quantification Algorithm
*In total 28 healthy Labrador Retriever dogs were sampled in a feed-challenge. The dogs are numbered 1-29 (nr 4 is missing). Fasting plasma samples were taken 15 minutes before serving of a test-meal (a-Fhr). Postprandial plasma samples were taken at 1 hour (b-1hr), 2 hours (c-2hr), 3 hours (d-3hr) and at 4 hours (e-4hr) after feeding.
