# Supplementary material for: Plasma metabolomics reveals lower carnitine concentrations in overweight Labrador Retriever dogs
Source: Acta Vet Scand. 2019 Feb 26;61:10. doi: 10.1186/s13028-019-0446-4 (PMC6390349; doi:10.1186/s13028-019-0446-4)
Supplement: Supplementary file 3 — Additional file 3. Evaluation of individual target metabolites quantified with the automated quantification algorithm (AQuA). [file 13028_2019_446_MOESM3_ESM.pdf]

Additional file 3.

Evaluation of individual target metabolites quantified with the Automated Quantification Algorithm (AQuA)

Metabolite	Position [a] (ppm)	Occurrence [b] (LOD)	Methodological variability [c]	Positional deviation [d]	Quality reached [e]
1,2-Propanediol	1.152	24	Medium/high	No	No
1-Methylhistidine	7.018	1	High	No	No
2-Aminobutyric acid	0.983	100	Low	No	Yes
2-Hydroxybutyric acid	0.903	100	Low	No	Yes
2-Hydroxyisovaleric acid	0.844	2	High	No	No
2-Ketoglutaric acid	3.012	100	Low	No	Yes
2-Oxoisocaproic acid	0.947	94	Low	No	Yes
2-Propanol	1.183	59	Medium	No	Yes
3-Hydroxybutyric acid	1.210	100	Low	No	Yes
3-Methyl-2-oxovaleric acid	0.898	80	Medium	No	Yes
3-Methylhistidine	8.065	83	Medium	No	Yes
Acetic acid	1.924	100	Low	No	Yes
Acetoacetic acid	2.285	99	Low/Medium	No	Yes
Acetone	2.237	100	Medium	No	Yes
Alanine	1.478	100	Low	No	Yes
Arginine	1.671	100	Low	No	Yes
Asparagine	2.946	100	Low	Yes	No
Betaine	3.906	100	Low	No	Yes
Carnitine	3.230	100	Low	No	Yes
Choline	3.206	100	Low	No	Yes
Citric acid	2.524	100	Low	No	Yes
Creatine	3.04	100	Low	No	Yes
Creatinine	4.064	100	Low	No	Yes
Dimethylglycine	2.930	100	Low	No	Yes
Dimethylsulfone	3.157	100	Low	Yes	No
Ethanol	1.189	86	High	No	No
Formic acid	8.459	100	Low	No	Yes
Glucose *	3.915	100	Low	No	Yes
Glutamic acid	2.361	4	High	No	No
Glutamine	2.479	100	Low	No	Yes
Glycerol	3.655	100	High	No	No
Glycine	3.567	100	Low	No	Yes
Histidine	7.095	100	Low	No	Yes
Isocaproic acid	0.879	92	Low	No	Yes
Isoleucine	1.019	100	Low	No	Yes
Lactic acid *	1.328	100	Low	No	Yes
Leucine	0.975	100	Low	No	Yes
Lysine	3.033	100	Low	No	Yes
Methanol	3.366	100	High	No	No
Methionine	2.647	100	Low	No	Yes
Myo-Inositol	4.073	100	Low	No	Yes
O-phosphocholine	3.223	100	Medium	Yes	No
Ornithine	3.061	99	Low	No	Yes
Phenylalanine	7.328	100	Low	No	Yes
Proline	4.150	100	Low	No	Yes
Propionic acid	1.060	14	High	No	No
Pyroglutamic acid	4.197	6	High	No	No
Pyruvic acid	2.379	100	Low	No	Yes
Sarcosine	2.743	100	Low	No	Yes
Serine	3.959	100	Low	No	Yes
Succinic acid	2.409	100	Medium	No	Yes
Threonine	4.258	100	Low	No	Yes
Trimethylamine N-oxide	3.269	100	Low	No	Yes
Tyrosine	7.205	100	Low	No	Yes
Valine	1.390	100	Low	No	Yes

[a] The chemical shift value used in AQuA.

[b] Signal-to-noise (S/N ratios) were calculated and a limit of detection (LOD) value of 3·Noise was set.

[c] Values are based on previously analysed quality control samples (Röhnisch HE *et al.* 2017). Coefficient of variation (CV) < 15 %: Low variability, CV 15-20%: Medium variability, CV > 20 %: High variability.

[d] Three metabolites had a positional deviation with overlap (Yes) of the target signal between experimental spectra, defined as a poor performance in deconvolution.

[e] Quantification quality were reached (Yes) in 43 of 55 targeted metabolites. Quality was based on: LOD ≥ 50%, a methodological variability ≤ 20% and an optimal performance in deconvolution. Of the 43 metabolites, 41 were included in the statistical analysis.

*These two metabolites have been excluded from the statistical analysis as their great variability suppressed the variation within the other metabolites in the multivariate statistical models.
