# Supplementary material for: Plasma metabolomics reveals lower carnitine concentrations in overweight Labrador Retriever dogs
Source: Acta Vet Scand. 2019 Feb 26;61:10. doi: 10.1186/s13028-019-0446-4 (PMC6390349; doi:10.1186/s13028-019-0446-4)
Supplement: Supplementary file 4 — Additional file 4. Principal component analysis score plots showing fasting and the 1–4 h postprandial metabolomes. [file 13028_2019_446_MOESM4_ESM.pdf]

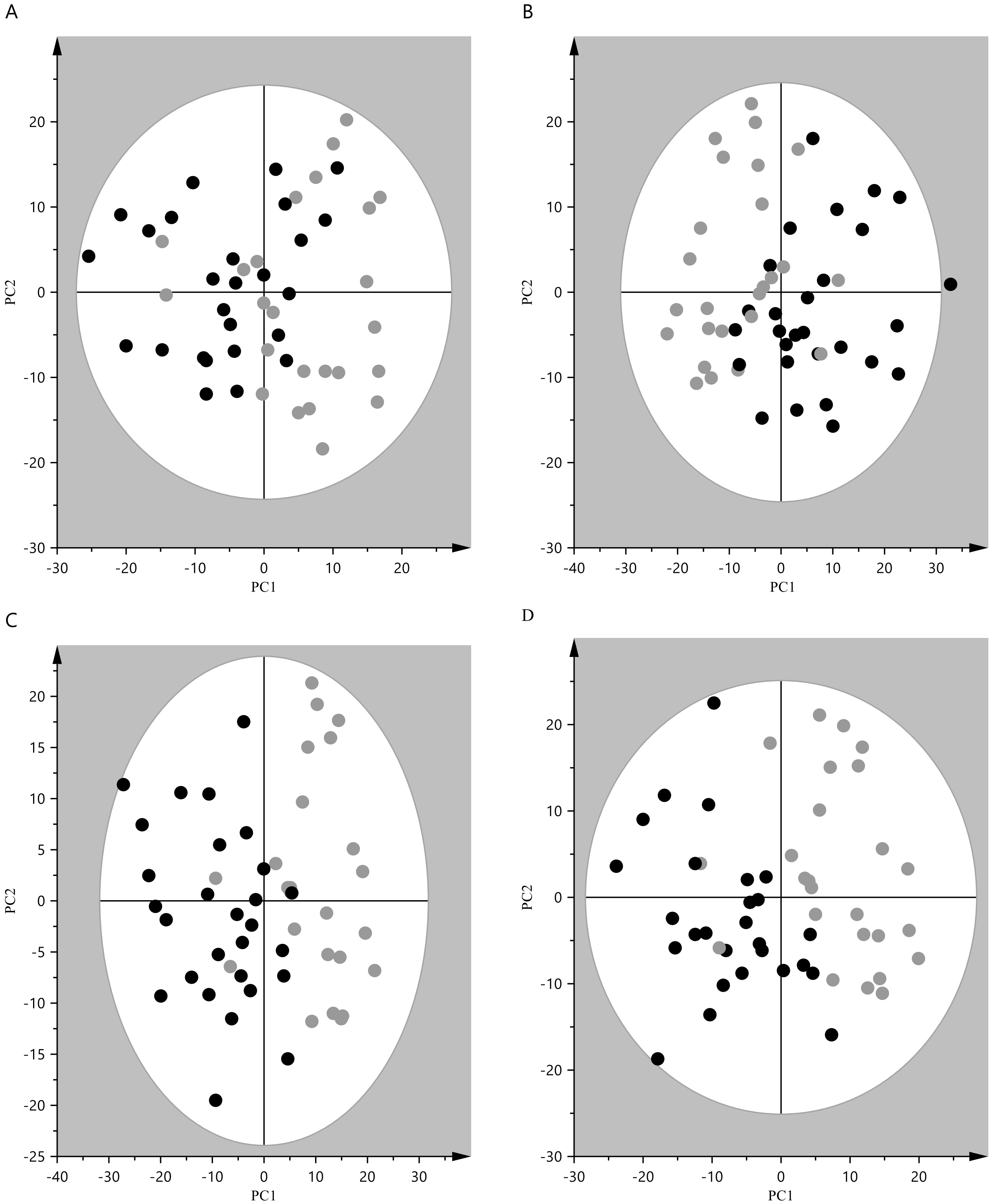

Additional file 4. Principal component analysis score plots showing fasting and the 1-4 hours postprandial metabolomes
Multivariate Principal component analysis (PCA) demonstrated clear visual separations between the fasting metabolome and all (1, 2, 3, and 4 hours) postprandial metabolomes. Four separate models were constructed (A-D) with all 28 dogs (regardless of body condition score) and 41 plasma metabolites included (the metabolites glucose and lactic acid were excluded from the statistical analysis as their great variability suppressed the variation within the other metabolites in the multivariate statistical models). The separations were significant in a partial least-squares discriminant analysis (PLS-DA) where fasting and 1-4 hours postprandial metabolomes, respectively, were pre-defined as separate groups (for *P*-values and discriminative metabolites; see Table 2.in Results section).

● Fasting metabolome

● Postprandial metabolome

(A) Fasting plasma metabolome and 1 hour postprandial metabolome. Principal component 1 (PC1) explained 11% of the total variance and PC2 8%.
(B) Fasting plasma metabolome and 2 hour postprandial metabolome PC1 explained 13% of the total variance and PC2 8%.
(C) Fasting plasma metabolome and 3 hour postprandial metabolome PC1 explained 13% of the total variance and PC2 7%.
(D) Fasting plasma metabolome and 4 hour postprandial metabolome PC1 explained 11% of the total variance and PC2 8%.
