# Supplementary material for: Plasma metabolomics reveals lower carnitine concentrations in overweight Labrador Retriever dogs
Source: Acta Vet Scand. 2019 Feb 26;61:10. doi: 10.1186/s13028-019-0446-4 (PMC6390349; doi:10.1186/s13028-019-0446-4)
Supplement: Supplementary file 5 — Additional file 5. Plasma ketone bodies in the meal-challenge test significant over time in the mixed model repeated measures analysis. [file 13028_2019_446_MOESM5_ESM.pdf]

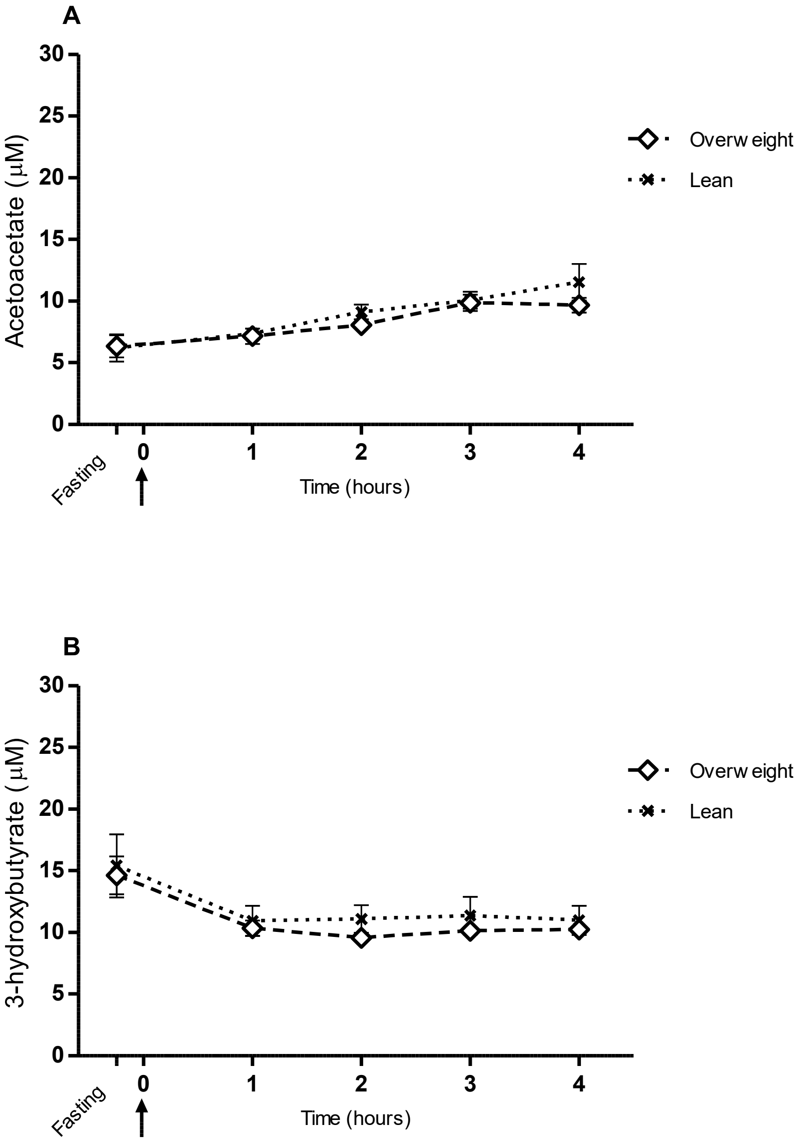

Additional file 5. Plasma ketone bodies in the meal-challenge test significant over time in the mixed model repeated measures analysis Plasma ketone bodies (A and B) significant over time in the feed-challenge test. Statistical analyses were conducted by a mixed model repeated measures analysis with Tukey-Kramer adjustment ($P<0.0001$ for both). Significance level $\alpha < 0.0012$ after Bonferroni corrections. Dogs were divided into body condition groups; lean (BCS 4-5, n=12) and overweight (BCS 6-8, n=16). Values are given as µM concentrations (mean ± SEM). Fasting plasma samples were taken 15 minutes before serving of a test meal at time 0 (arrow). Ketone concentrations in lean and overweight dogs are shown as response curves from fasting to 4 hours after feeding. No significant differences in overall responses with regards to body condition groups were found (A and B). Logarithmical transformation for 3-hydroxybutyrate was applied before the statistical analyses.
