# Supplementary material for: Plasma metabolomics reveals lower carnitine concentrations in overweight Labrador Retriever dogs
Source: Acta Vet Scand. 2019 Feb 26;61:10. doi: 10.1186/s13028-019-0446-4 (PMC6390349; doi:10.1186/s13028-019-0446-4)
Supplement: Supplementary file 6 — Additional file 6. The sulfur amino acid methionine analysed by the mixed model repeated measures in the meal-challenge test. [file 13028_2019_446_MOESM6_ESM.pdf]

A

Metabolite	Fasting	Postprandial			
	Mean ± SD (µM)	Hours	Mean ± SD (µM)	*P*-value[b]	SE[c]
Methionine	42.7±7.8	1	42.5±6.5	1.00	0.62
		2	42.9±6.6	0.98	0.85
		3	42.0±5.3	0.98	1.00
		4	41.6±4.8	0.93	1.13

B

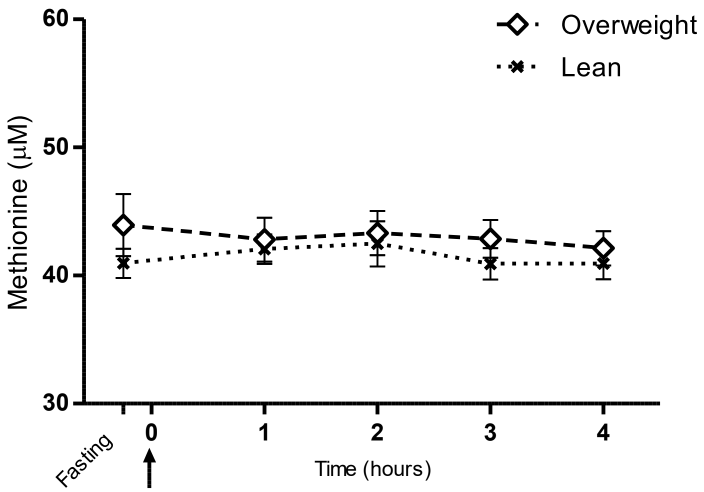

Additional file 6. The sulfur amino acid methionine analysed by the mixed model repeated measures in the meal-challenge test. Mixed model repeated measures analysis with significance level *P*<0.05 and Tukey-Kramer adjustment within the model.

A) Fasting and postprandial methionine concentrations (µM) are shown as mean ± standard deviation (SD). *P*-values for comparisons between fasting and each postprandial time point and standard error (SE) for comparisons between fasting and postprandial time points, respectively. All 28 dogs included.

B) Dogs were divided into body condition groups; lean (BCS 4-5, n=12) and overweight (BCS 6-8, n=16). The postprandial methionine response was not significant over time nor between body condition groups. Values are given as µM concentrations (mean ± SEM). Fasting plasma samples were taken 15 minutes before serving of a test meal at time 0 (arrow).
